# Global Reorganization of the Nuclear Landscape in Senescent Cells

**DOI:** 10.1016/j.celrep.2014.12.055

**Published:** 2015-01-29

**Authors:** Tamir Chandra, Philip Andrew Ewels, Stefan Schoenfelder, Mayra Furlan-Magaril, Steven William Wingett, Kristina Kirschner, Jean-Yves Thuret, Simon Andrews, Peter Fraser, Wolf Reik

**Affiliations:** 1 Epigenetics Programme, The Babraham Institute, Cambridge CB22 3AT, UK; 2 The Wellcome Trust Sanger Institute, Cambridge CB10 1SA, UK; 3 Nuclear Dynamics Programme, The Babraham Institute, Cambridge CB22 3AT, UK; 4 Cambridge Institute for Medical Research, University of Cambridge, Cambridge CB2 0XY, UK; 5 CEA, iBiTec-S, SBIGeM/CNRS FRE3377 I2BC/Université Paris-Sud, Gif-sur-Yvette 91191, France; 6 Bioinformatics Group, The Babraham Institute, Cambridge CB22 3AT, UK

## Abstract

Cellular senescence has been implicated in tumor suppression, development, and aging and is accompanied by large-scale chromatin rearrangements, forming senescence-associated heterochromatic foci (SAHF). However, how the chromatin is reorganized during SAHF formation is poorly understood. Furthermore, heterochromatin formation in senescence appears to contrast with loss of heterochromatin in Hutchinson-Gilford progeria. We mapped architectural changes in genome organization in cellular senescence using Hi-C. Unexpectedly, we find a dramatic sequence- and lamin-dependent loss of local interactions in heterochromatin. This change in local connectivity resolves the paradox of opposing chromatin changes in senescence and progeria. In addition, we observe a senescence-specific spatial clustering of heterochromatic regions, suggesting a unique second step required for SAHF formation. Comparison of embryonic stem cells (ESCs), somatic cells, and senescent cells shows a unidirectional loss in local chromatin connectivity, suggesting that senescence is an endpoint of the continuous nuclear remodelling process during differentiation.

## Highlights

• SAHF cells show sequence- and LAD-dependent loss of heterochromatin (HC) structure
• Senescence HC behavior is mirrored in Hutchinson-Gilford progeria
• Senescence-specific spatial clustering of HC leads to a new model for SAHF formation
• Comparing ESCs, somatic, and senescent cells links senescence to differentiation

## Graphical Abstract

**Figure:**
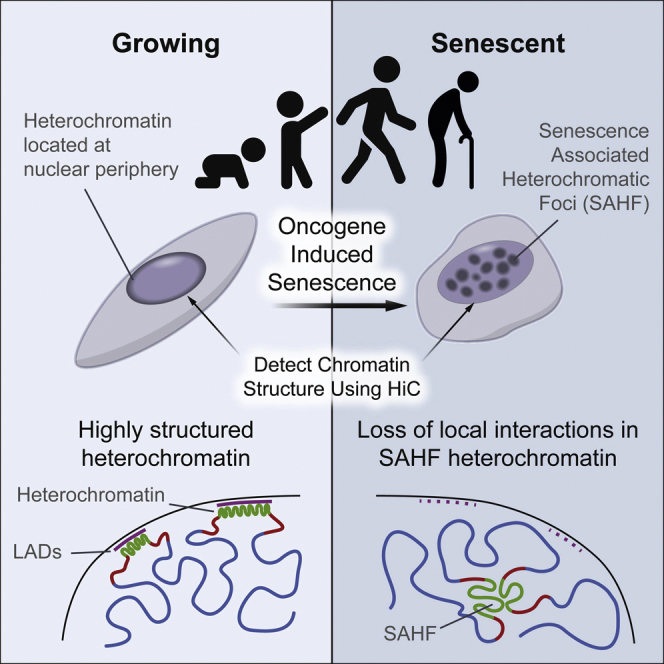

## Teaser

Chandra, Ewels, et al. map changes in genome organization in cellular senescence using Hi-C. Contrary to the believed increase in heterochromatin in senescence-associated heterochromatic foci formation, they describe a loss of local interactions in heterochromatic regions. This is in agreement with changes observed in progeria cells.

## Introduction

Cellular senescence is an irreversible cell-cycle arrest, originally described for primary cells after long-term cell culture and attributed to telomere attrition (Hayflick and Moorhead, 1961). More recently, cellular senescence has been established as a cellular response to a variety of stresses such as DNA double-strand breaks or oncogene activation (Di Leonardo et al., 1994; Lin et al., 1998; Serrano et al., 1997).

Oncogene-induced senescence (OIS) is an intrinsic tumor suppressor mechanism, involving activation of the key tumor suppressor pathways p53 and pRB/p16INK4a. Inactivation of one or both of these pathways is found in the majority of cancers. Markers of senescence, such as p16 upregulation, are particularly prevalent in benign lesions and are often lost upon malignancy (Braig et al., 2005; Haugstetter et al., 2010; Michaloglou et al., 2005). Reactivation of p53 in mouse models of liver cancer leads to senescence with subsequent immune clearance of cancer cells (Xue et al., 2007). A key aspect of the senescence response implicated in the immune clearance is the senescence-associated secretory phenotype (SASP) (Acosta et al., 2008; Coppé et al., 2008; Kuilman et al., 2008). SASP is characterized through the secretion of cytokines, which are able to induce paracrine senescence in neighboring cells (Acosta et al., 2013). Recent work has implicated cellular senescence in normal developmental processes (Muñoz-Espín et al., 2013; Storer et al., 2013).

In addition to its role in oncogenesis, a role for senescence in organismal aging has recently been substantiated; the depletion of senescent cells has been shown to relieve symptoms in mouse models of age-related diseases, suggesting that cellular senescence may be a useful model system for organismal aging (Baker et al., 2011; López-Otín et al., 2013).

Previous work has shown that cellular senescence in human diploid fibroblasts is accompanied by a large-scale spatial rearrangement of chromatin, forming nuclear structures known as senescence-associated heterochromatic foci (SAHF). SAHF are enriched in constitutive heterochromatic markers, such as H3K9me3 and HP1 proteins (Narita et al., 2003). However, SAHF formation does not occur in all senescent cells. The proportion of cells exhibiting SAHF depends on the method of senescence induction, ranging from a few percent in replicative senescence to nearly 90% in c-raf OIS (Jeanblanc et al., 2012). In contrast, other cellular models of organismal aging such as cells from Hutchinson-Gilford progeria syndrome (HGPS) patients show a decrease in heterochromatin and are devoid of SAHF (Scaffidi and Misteli, 2006; Shumaker et al., 2006). Cellular models of HGPS and cellular senescence of fibroblasts have proven to be relevant models for organismal aging. It is therefore important to understand the seemingly contradictory roles of heterochromatin in cellular aging and SAHF formation.

We have recently shown that SAHF chromosomes show an inversion of euchromatin, facultative heterochromatin (fHC), and constitutive heterochromatin (cHC), with cHC moving to the center of chromosomal territories (see also Figure 4C; Chandra et al., 2012). This inversion is due to a physical reorientation of the chromatin rather than a redistribution of repressive histone marks, questioning a causal role for classical heterochromatic marks, H3K9me3 (cHC) and H3K27me3 (fHC), in the formation of heterochromatin in somatic cells. A key feature of the senescent nucleus and strong correlate with SAHF formation is the loss of lamin B1 (Sadaie et al., 2013; Shah et al., 2013). Other factors involved in SAHF formation, such as the cell-cycle regulator pRb, high mobility group proteins HMGA1/HMGA2, histone chaperones HIRA and ASF1a, canonical Wnt signaling, chromatin remodelling proteins p400 and BRG1, and linker histone H1, have been identified; however, knowledge of how the chromosome structure is changed is still lacking (Chan et al., 2005; Chicas et al., 2010; Funayama et al., 2006; Narita et al., 2003, 2006; Tu et al., 2013; Ye et al., 2007a, b; Zhang et al., 2005). More importantly, the function of SAHF is controversial. Whereas the role of SAHF was initially reported as being tumor and cell-cycle suppressive (Narita et al., 2003, 2006), recent work has suggested that SAHF may in fact be proproliferative (Di Micco et al., 2011).

To gain insight into the function of SAHF, we decided to unravel the physical structure of senescent chromatin in unprecedented detail, combining fluorescence in situ hybridization (FISH) with Hi-C to map the physical changes that accompany SAHF formation. We find dramatic changes in both the global interaction network and local neighborhood of genomic regions. Surprisingly, we find distinct global changes in the interactions and compaction of certain classes of lamin-associated domains, defined by continuous genomic fragments of homogenous guanine-cytosine (GC) content (isochores; Bernardi, 2012). Contrary to the current view of enhanced heterochromatinization in SAHF formation, we find a loss of internal structure in constitutive heterochromatic (cHC) regions in cellular senescence. This loss of internal structure is accompanied by spatial clustering of the cHC regions. We further show that HGPS cells behave similarly to senescent cells in their local interaction changes but do not exhibit the spatial clustering of cHC, suggesting a two-step mechanism for SAHF formation. Finally, we investigate embryonic stem cells (ESCs) and senescent cells and find a fundamentally opposing local architecture with somatic cells representing an intermediate state.

## Results

### Senescence Causes Global Shifts in Local Chromatin Interactions

**Figure 1:**
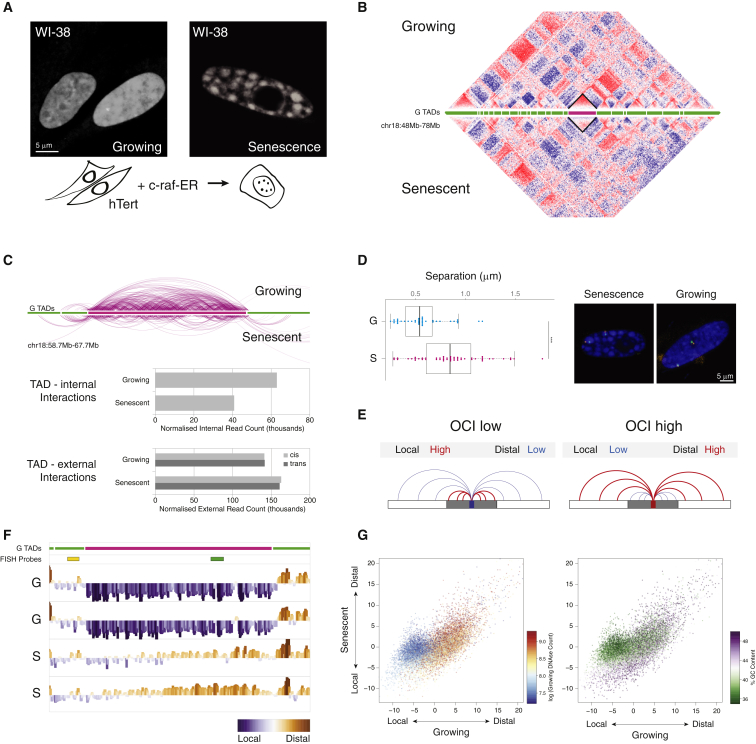
Senescence Is Accompanied by Local and Global Changes in the Interaction Pattern of the Genome (A) WI-38 hTERT/GFP-RAF1-ER cells show the characteristic SAHF phenotype upon senescence induction. (B) Interaction heatmaps for chr18q for growing (top) and senescence (bottom). Strong interactions are shown in red whereas weak interactions are in blue. Green lines (center) represent topologically associating domains (TADs) determined in growing cells. Highlighted TAD (black rhombus and pink TAD line) suggests a loss of internal interactions. (C) Top: genome browser shot of interactions reaching into and from within the depicted TAD. Growing cells show more TAD internal interactions than senescent cells. Bottom: normalized read counts from highlighted TAD. Internal contacts confirm loss of TAD internal interactions and external interactions increase in senescence, both in *cis* (light gray) and in *trans* (dark gray). (D) DNA-FISH showing separation between probes located within and adjacent to the highlighted TAD. Separation increases in senescence p = 0.00003 (Mann-Whitney-Wilcoxon test). Shown to the right are representative confocal microscopy planes of the FISH separation experiment. (E) Top: schematic of the open chromatin index (OCI), which describes regions changing their ratio between local and distal contacts. (F) Browser shot of OCI in two biological replicates (G, growing; S, senescence) over highlighted TAD (pink) shows a loss of local interactions. (G) Scatterplots comparing genome-wide OCI in 200 kb windows between growing and senescence. Points are colored by DNase accessibility as measured in growing fibroblasts (left) and %GC content (right). A cluster of points can be seen to deviate from the diagonal, which shows a loss of local contacts in senescence. These regions are the least accessible in the genome and have a low GC content.

To study OIS, we used the WI-38hTERT/GFP-RAF1-ER model system (Jeanblanc et al., 2012). Senescence is induced by the activation of the GFP-RAF1-ER kinase by addition of 4-hydroxy-tamoxifen. The nuclear phenotype in these cells is highly homogenous compared to other OIS systems such as Ras-induced senescence, with a high percentage of SAHF-positive cells (∼86%; Figures 1A and S1A). Analyses of bromodeoxyuridine (BrdU) incorporation and abundance of key senescence markers at 48 hr after induction confirmed an OIS phenotype (Figures S1A and S1B).

Previous work on the reorganization of chromatin in senescence has revealed a complex change in nuclear architecture; intrachromosomal heterochromatin clusters within SAHF without redistribution of repressive histone marks along the linear DNA sequence (Chandra et al., 2012; see also Figure 4C). These observations suggest a dynamic remodelling of the nucleus driven by spatial changes, as opposed to a change in chromatin identity (Chandra and Narita, 2013). To further understand these changes in nuclear architecture in growing and senescent cells, we performed Hi-C, a proximity-based ligation assay that measures the frequency of close chromatin interactions, as summarized in Figure S1C (Lieberman-Aiden et al., 2009). Two independently grown and fixed batches of cells were sequenced to a depth of at least 114 million reads. Data were processed using the HiCUP pipeline (S.W.W. and S.A., unpublished data; Figure S1D). For initial inspection, we plotted Hi-C interaction heatmaps using HOMER (Figure 1B; Heinz et al., 2010). The heatmaps show a typical pattern of self-interacting topologically associated domains (TADs) (Dixon et al., 2012; Nora et al., 2012). Although the global chromosomal interaction pattern appears largely unchanged between growing and senescent cells, closer inspection shows striking differences in the strength of internal interactions within TADs (Figures 1B and 1C). To further investigate this phenomenon, we calculated the number of interactions in which both interacting regions are located within a single TAD versus those spanning the TAD boundaries (Figure 1C). This revealed a loss of interactions within the TAD and a corresponding gain of cross-boundary interactions in senescent cells (Figure 1C). To assess the consequences of loss of local contacts for chromatin compaction, we measured the distance between two FISH probes (separated by ∼2.2 Mb)—one approximately 100 kb upstream of the TAD and the corresponding probe inside the TAD (Figures 1D and 1F for probe locations). We found a substantial shift in the mean distance separating the probes from 0.57 μm in growing to 0.86 μm in senescent cells (Wilcoxon-Mann-Whitney test; p = 0.00003). This observation confirms for this TAD that a loss of local interactions leads to a change in physical compaction in senescence.

To study this change in local interaction frequency more precisely, we developed a measure to examine the difference in local versus distal interactions genome wide, which we call open chromatin index (OCI) (Figure 1E). This analysis counts the number of interactions with both Hi-C reads within a window and compares these to a control. Initially, we used interaction counts across the remainder of the chromosome as a control but found that results were biased due to the varying lengths of chromosomes. As an alternative, we used the number of interchromosomal contacts. The distal and interchromosomal interaction counts behaved concordantly (Figure 1C), and the latter provided a more stable metric. We measured the ratios of these interactions in windows across the genome and normalized by subtracting the median chromosomal value from each window and then by smoothing values in a rolling 20 Mb window. The resulting OCI values give insight into the propensity of a region to form local or distal interactions. Our measurement bears some resemblance to the previously described interchromosomal contact propensity (ICP) (Kalhor et al., 2012), which itself has been found to correlate with active marks such as RNA polymerase II occupancy (Kalhor et al., 2012). When visualized over the TAD shown in Figures 1B and 1C, we find a rise in the OCI in senescence, suggesting OCI may be a suitable metric to measure a switch from local to distal interactions (Figure 1F). To explore OCI changes genome wide, we plotted OCI values in growing and senescence in a scatterplot (Figure 1G). A population of regions appears to follow the behavior of the TAD described above: an initially low OCI in growing cells with a rise in senescent cells, indicating a loss of local interactions. It has been previously suggested that high ICP values could be affected by proximity to the periphery of chromosomal territories (Kalhor et al., 2012). To rule out any such effect in our measurements, we calculated the OCI using only *cis* contacts, counting any interaction spanning more than 20 Mb as a distal contact (Figures S1E and S1F). We readily identified the same changing regions, confirming OCI as a suitable metric to measure changes in local architecture.

Based on the TAD shown in Figures 1B, 1C, and 1F, a low OCI in growing cells would suggest a compact structure with strong local interactions. To test this hypothesis, we correlated OCI with DNase accessibility in growing cells (Figure 1G). Corroborating our hypothesis, we found a striking overlap between the least-accessible regions in growing cells (dark blue) and regions showing the strongest rise in OCI. Next, we analyzed whether genomic regions with changing chromosomal interactions display a characteristic sequence-composition signature. We highlighted the GC content of each point within the ICP scatterplot and again identified a strong correlation, with those regions losing internal contacts strongly enriched for low GC content (Figure 1G).

Using OCI, we have identified regions of chromatin losing internal structure. These regions are the least accessible in the genome in growing cells and are rich in adenine-thymine (AT) content.

### Sequence Composition Predicts Structural Chromatin Dynamics in Senescence

The bias in the GC content of regions showing changing OCI (Figure 1G) led us to investigate the role of sequence composition within senescent nuclear reorganization. We used a recently published annotation to split the genome into isochores, large blocks of similar GC content (in GC%: L1 <37; L2 37–41; H1 41–46; H2 46–53; and H3 >53), which show little compositional heterogeneity (Costantini et al., 2006). Isochores were originally resolved according to their behavior in density gradient centrifugation, adding a dimension of macromolecular behavior to our analysis of sequence content (Macaya et al., 1976; Thiery et al., 1976).

**Figure 2:**
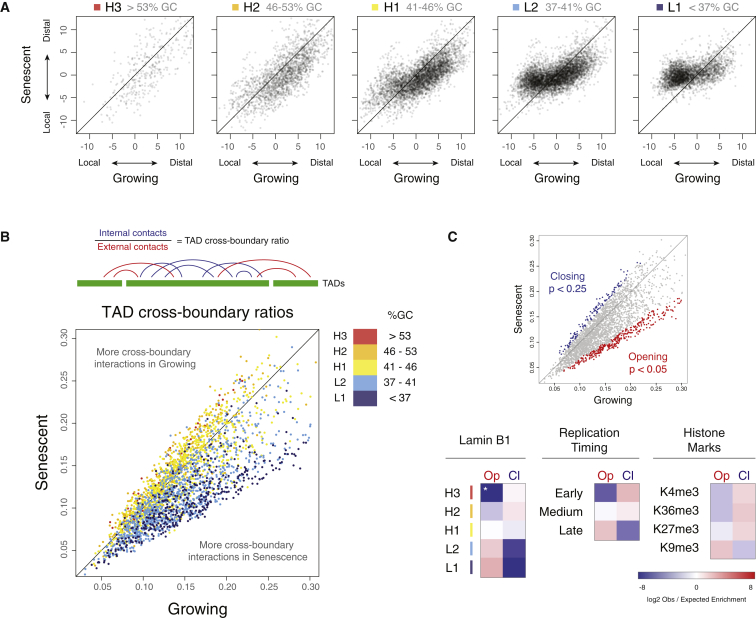
Sequence Composition Predicts Structural Chromatin Dynamics in Senescence (A) Scatterplots showing OCI calculated in 200 kb windows for growing and senescence cells, separated by overlap with isochores. The greatest changes can be seen to occur in the L1 isochores. (B) Top: schematic of TAD boundary strength calculation. Bottom: scatterplot comparing the cross-boundary ratios over TADs genome wide, colored by isochore. The L-isochores can be seen to gain cross-boundary interactions in senescence. (C) Top: selection of opening and closing TADs highlighted in scatter plot (a less-stringent cutoff was chosen for closing TADs, in order to reach a comparable number). Bottom: enrichment for overlap with genomic features (log2 obs/exp; also see Experimental Procedures) for opening (Op) and closing (Cl) TADs.

To see how isochores behave in growing and senescent cells, we plotted the OCI for each isochore (Figures 2A and S2A). We observe a large difference in the behavior between L-isochores (L1 and L2) and H-isochores (H1, H2, and H3), with the majority of regions losing their local interactions within the GC-poor isochores (Figure 2A). Our data extend the role of the sequence composition in higher-order chromatin dynamics in senescence to the physical entities of isochores.

TADs are self-interacting domains with boundaries defined by a change in the directionality bias of interacting fragments. We calculated the position of TAD boundaries within growing and senescent cells, as previously described (Dixon et al., 2012). We found a high 89.3% percent of the domains to be conserved between growing and senescent cells.

TAD boundary strength and decreased TAD internal contacts have been shown to be affected by acute loss of a functional cohesion complex (Sofueva et al., 2013). Interestingly, a recent report suggests a loss of CTCF at *CDKN2A* in oncogene-induced senescence (Hirosue et al., 2012). To test whether TAD boundaries are affected by a change in OCI, we calculated the ratio of interactions found within TADs versus those spanning a boundary (Figure 2B). We found a change in insulation strength that correlates with isochores. L1 TAD boundaries show a striking loss of boundary strength (“opening”), whereas H2 and H3 TAD boundaries appear to strengthen slightly (“closing”; Figures 2B and 2C). Thus, whereas the position of TAD boundaries remains largely unchanged in senescent cells, their quality is affected by changes in local and distal interactions, with a significant fraction of TADs losing insulation strength and a smaller fraction gaining insulation strength.

To test the changing TADs for enrichment in genomic features, we selected regions based on the significance of the difference in insulation strength (p < 0.05 for opening TADs; p < 0.25 for closing TADs; Figure 2C). We find opening TADs are enriched in H3K9me3, late replication timing (RT), and lamina-associated domains (LADs). TADs with stronger boundary insulation show a strong underrepresentation in these features and are instead enriched in regions of early replication and H3K36me3.

### Sequence Composition and Lamin Association Predict OCI Increase in Senescence

Recent research has highlighted the importance of LADs and the loss of LMNB1 for the senescence phenotype (Sadaie et al., 2013; Shah et al., 2013; Shimi et al., 2011). The level of LMNB1 reduction was shown to predict SAHF-positive cells in a heterogeneous population, and ectopic expression of LMNB1 was able to reduce the number of SAHF-positive cells (Sadaie et al., 2013). L1 isochores showed the strongest loss of local interactions (Figure 2A) and TAD isolation strength (Figure 2B) and were strongly enriched in LADs (Figure 2C). To understand the behavior of LADs, we plotted the change in average OCI across LADs (Figure S2B). We find a strong correlation between isochore class and LAD OCI behavior, with L1 LADs losing and H-LADs gaining local interactions. These observations allow us to predict LAD behavior by sequence content alone.

**Figure 3:**
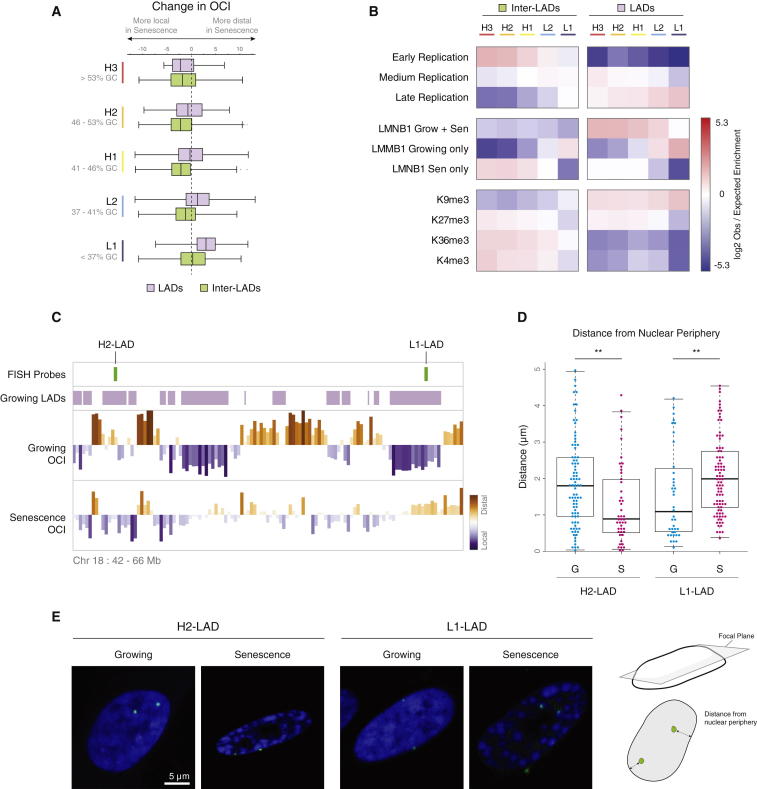
Combined Sequence Composition and Lamin Association Predict the Strongest OCI-Gaining Regions, which Are Changing Their Nuclear Positioning (A) Change in OCI levels in senescence for regions overlapping LADs (purple) and inter-LADs (iLADs, green). (B) Enrichment of LADs and iLADs for replication timing, LMNB1 regions, and several chromatin marks. (C) Browser shot indicating genomic location for FISH probes designed against two adjacent H2-LAD and L1-LAD regions (green vertical lines). (D) Distances of FISH signals to the nuclear periphery. (E) Left: representative confocal microscopic images of FISH-treated growing and senescent cells. Right: schematic showing the measurements made for the DNA-FISH from the central focal plane.

To exclude the possibility that this observation is due only to the enrichment of LADs within AT-rich regions of the genome, we calculated OCI changes across LAD and inter-LAD regions of each isochore (Figure 3A). We find that the loss of internal contacts for L-isochores (a rise in OCI) is LAD dependent, with L-inter LADs showing no change in OCI. This suggests that the combination of being in an AT-rich L-isochore and a LAD predicts the dramatic OCI gain in senescence. For H2 and H3 isochores, the OCI change is not dependent on lamin binding (Figure 3A), suggesting that other features control the behavior of these LADs.

Senescence is accompanied by a major loss of LMNB1, and recent chromatin immunoprecipitation sequencing (ChIP-seq) experiments have identified the majority of the regions losing LMNB1 occupancy in senescence (Sadaie et al., 2013). Despite this dramatic loss, a subset of regions not bound by LMNB1 in growing cells appears to gain binding in senescence (Sadaie et al., 2013). We calculated the enrichment of these growing- and senescent-specific LMNB1 regions across the isochore LADs and inter-LAD regions (Figure 3B). We observe a strong enrichment for areas losing LMNB1 in L-isochore lamin-associated domains (L-LADs), suggesting the loss of LMNB1 may be involved in the architectural changes we have uncovered. There was no enrichment of LMNB1-gaining regions in any LAD category, supporting the previous observation that these regions are unique to senescence. GC-rich inter-LADs were enriched for senescence-specific LMNB1 binding, showing the opposite isochore pattern to the loss of lamin. We tested the overlap between opening and closing TADs and growing/senescence-specific LMNB1 regions (Figure S3A). We find a strong enrichment for the newly forming senescence-specific LADs within TADs exhibiting greater insulation, suggesting a functional role for the senescence-specific gain of LMNB1. Our data suggest a role for the chromosomal redistribution of LMNB1 in the architectural changes we described.

To investigate whether the changes in LAD OCI are accompanied by changes in positioning in the nucleus, we used DNA-FISH microscopy to measure the distance of specific LADs to the nuclear periphery. We designed probes within two adjacent L1- and H2-LADs (Figure 3C); the L1-LAD showed a significant increase in the mean distance to the periphery (Figures 3D and 3E; p < 0.006). Interestingly, the H-LAD also showed a significant change, moving closer to the periphery (Figures 3D and 3E; p < 0.009). We tested another L1-LAD in close proximity to the *CDKN2A* locus and observed a similar move away from the nuclear periphery (Figure S3B; p < 0.002). These observations suggest that the consequences of chromatin restructuring go beyond the change in local interactions described by OCI and could additionally entail a change in nuclear positioning, with L1 LADs moving away from the periphery and H-LADs moving closer to it.

### Local Changes Are Accompanied by the Formation of Specific Distal Interactions

**Figure 4:**
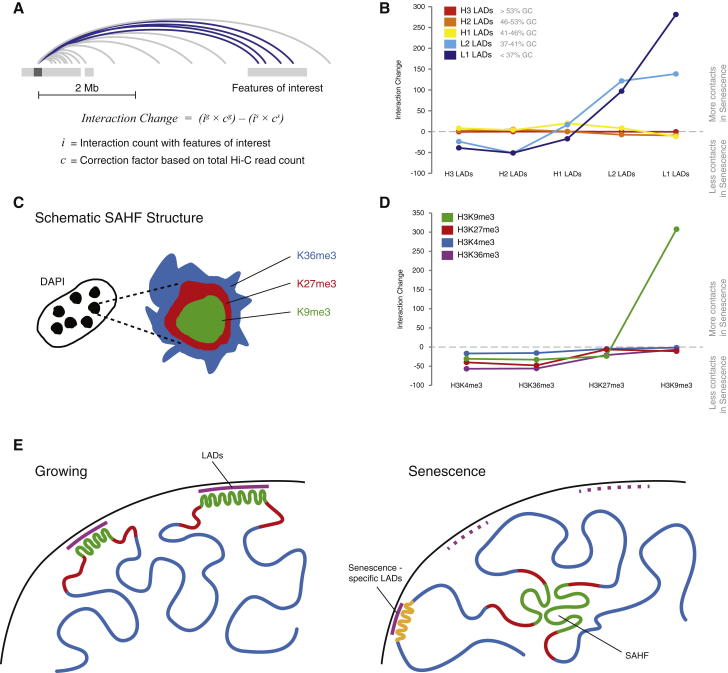
Local Changes Are Accompanied by the Formation of Specific Distal Interactions (A) Schematic showing how the change in interaction strength between features was calculated. *g* and *s* supertext denotes growing and senescence. Note the avoidance of features within 2 Mb. (B) Change in interaction strength calculated between isochore LADs comparing growing and senescence (C) Schematic depicting the chromatin organization of histone marks in SAHF as described in Chandra et al. (2012). (D) Change in interaction strength calculated between regions associated with histone marks. (E) Model depicting the change of the chromatin architecture. We propose that L-LAD regions with strong local interactions (high OCI) in growing (green) detach from the nuclear lamina and lose their internal structure (potentially forming the core of the SAHF). Other regions, such as selected H-LADs, move toward the nuclear periphery.

Through changes in OCI and TAD insulation strength, we observe a redistribution of local interactions to more distal interactions for L-LADs. To test whether these new distal contacts are distributed randomly or form specific interactions, we mapped the change in normalized interaction count between different LADs. We calculated all reads linking selected features while ignoring interactions separated by less than 2 Mb to avoid any distance effects (Figure 4A). We observe a strong increase in the number of interactions between L-LADs, with the strongest gain found between L1-LADs (Figure 4B). In contrast, we do not observe any major changes in interactions between H-LADs.

Whereas L-LADs lose internal contacts, they increase their interactions between each other across the genome, indicating that they cluster together in nuclear space. This observation is reminiscent of the rearrangement of heterochromatin observed with microscopy during SAHF formation in Ras-induced senescence (Figure 4C; Chandra et al., 2012). We therefore analyzed whether interactions between domains defined by specific histone marks change in senescence. Consistent with previous microscopy data, we find that H3K9me3 domains come together in a specific manner during senescence (Figure 4D).

The concerted local loss of internal contacts and global clustering of L-LADs leads us to the model depicted in Figure 4E, whereby lamin-bound regions detach from the nuclear periphery and cluster within the nuclear interior. Some GC-rich H2/H3 regions appear to have gained internal structure and relocate to the periphery, consistent with a scenario of nuclear reshuffling resulting from LAD relocation.

Analyzing distal contacts of regions dramatically changing their local structure (L1-LADs and H3K9me3 domains) suggests a clustering of these regions within the nucleus. Previous work has speculated that SAHF may represent a special heterochromatic compartment, supported by immunofluorescence studies using DNA stains such as DAPI which show a very high intensity signal within the SAHF core. Contrary to this theory, our initial observations using Hi-C data suggest a loss of internal structure and possible decompaction of DNA enriched in the SAHF core. The clustering shown here can now explain the perceived expansion and clustering of heterochromatin and provides a new depth of understanding of the structural organization of senescent chromatin.

### Pre-senescent Replication Timing Predicts Chromatin Changes

OIS shows an accumulation of cells arrested in S phase. This has been linked to replication-stress-induced DNA damage signaling, possibly caused by an initial hyperproliferative burst following oncogene activation (Bartkova et al., 2006; Di Micco et al., 2006). Although the relationship between structural chromatin changes and cellular senescence is not well understood, one key protein required for SAHF formation is pRB, linking higher-order chromatin changes and replication (Narita et al., 2003). The interplay between replication timing and SAHF architecture has also been shown by pulse-chase studies, tracking nucleoside analogue incorporation (Chandra et al., 2012).

**Figure 5:**
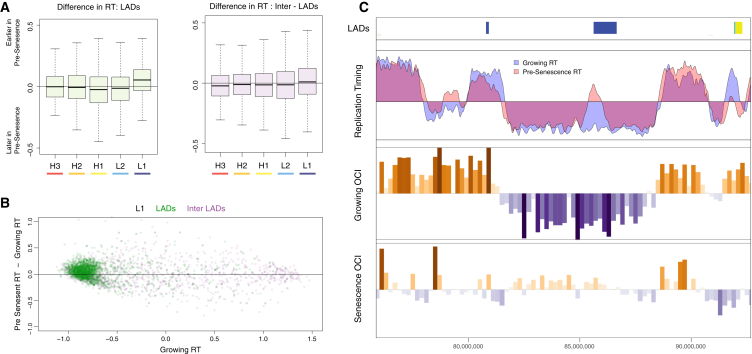
Presenescent Replication Timing Predicts Chromatin Changes (A) Change in replication timing between growing OIS ER-Ras cells and presenescent cells. Regions overlapping LADs are shown in green and iLADs in purple, divided by isochore. (B) Scatterplot showing difference between presenescent RT and growing RT versus growing RT for isochore L1; LADs (green) and iLADs (purple) are highlighted. Points above the horizontal are replicating earlier in presenescent cells. (C) Browser shot showing a L1-LAD region changing in presenescent RT.

Having identified the regions with the most dynamic structural changes in the Hi-C data, we tested whether these regions show any change in pre-senescence replication timing (RT). We reanalyzed whole-genome replication timing data from IMR90 ER-Ras cells, comparing growing cells and presenescent cells (48 hr after induction of Ras; Chandra et al., 2012). SAHF formation takes longer in ER-Ras cells, and kinetics of senescence establishment are better described, suggesting that changes observed at 48 hr would precede SAHF formation (Young et al., 2009). To compare the two data sets, we measured the change in normalized replication timing (Figure 5A). We split the genome into LADs and inter-LADs and then isochores. We see a shift toward earlier RT for L1-LADs, whereas L1 iLADs show little change. The scatterplots for the L1 isochore indicates a split between LADs (green) and iLADs (purple) into late and early replication timing, respectively (Figure 5B). A subpopulation of L1 LADs appears to be biased toward earlier RT in presenescence. An example of a locus exhibiting this shift can be seen in Figure 5C, which shows a region surrounding an L1-LAD losing its internal contact bias and changing in presenescence RT.

We find a correlation between the most significant structural changes in senescence as revealed by Hi-C and RT changes in hyperproliferating presenescent cells. This observation may indicate a role for replication timing in shaping the senescence chromatin landscape.

### Higher-Order Chromatin Dynamics in Senescence Reflect Changes in Progeria and May Represent the Endpoint of a Continuous Remodelling Process in Differentiation

The role of p16-dependent senescence in organismal aging highlights the importance of cellular senescence as an in vitro aging model (Baker et al., 2011). Fibroblasts belonging to patients suffering from Hutchinson-Gilford progeria syndrome (HGPS) undergo premature senescence and also provide an important model to study aging. However, OIS and HGPS cells have distinct chromatin features; for example, SAHF formation is exclusive to OIS. Whereas OIS is accompanied by a major loss in LMNB1, premature aging in HPGS is due to the accumulation of progerin, a mutated version of LMNA/LMNC (Eriksson et al., 2003). As such, a commonality found within both models is the destabilization of the nuclear lamina. To investigate the breadth of our findings regarding local chromatin changes in senescence and to examine similarities between OIS and HGPS fibroblasts, we compared our data to recently published Hi-C data sets in HGPS fibroblasts (McCord et al., 2013).

**Figure 6:**
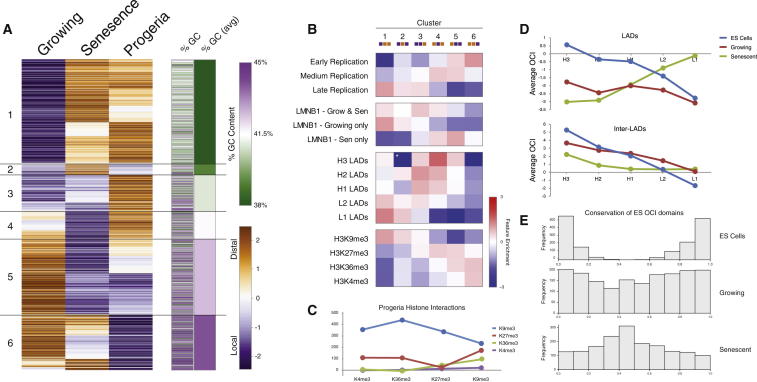
Higher-Order Chromatin Dynamics in Senescence Reflect Changes in Progeria and May Represent the Endpoint of a Continuous Remodelling Process in Differentiation (A) Hierarchical clustering of OCI values in 1 Mb of genomic windows for growing, senescence, and progeria. Columns to the right show GC content per genomic window and the average %GC per cluster. (B) Enrichment of clusters over genomic features. Note that clusters 1 and 5 show similar behavior in growing and senescence. (C) Change of interactions calculated between regions associated with histone marks. Progeria shows no clustering of H3K9me3 regions or other histone marks compared to growing cells. Enrichment calculated as shown in Figure 4A. (D) Average OCI over LADs (top) and iLADs (bottom) split by isochore in ESCs, growing, and senescence. (E) Conservation of OCI domains called using a hidden Markov model (see Experimental Procedures) in ESCs. The x axis shows percentage of windows within domains classed as local (0) or distal (1). ESCs show a bimodal distribution as expected. Growing and senescent cells show a decaying conservation of interaction state within these domains.

Unsupervised hierarchical clustering of OCI in growing, senescent, and progeroid fibroblasts split the data into six classes, which show strong correlation with GC content (Figure 6A). Classes 1 and 5 show similar trends for senescence and progeria and represent 58% of the genome (Figure S4). Class 1 is GC poor and highly enriched in L1-LADs (Figure 6B), suggesting a common loss of internal structure for these regions in both senescence and progeria. Cluster 5 shows the strongest enrichment of all clusters for regions gaining LMNB1 in senescence, suggesting that a common mechanism may lead to the compaction of these regions.

Whereas the decompaction of heterochromatin for HGPS cells is consistent with previous observations (Scaffidi and Misteli, 2006; Shumaker et al., 2006), the congruency in changing OCI seems to contradict the fact that HGPS cells do not form SAHF, suggesting that changes in OCI are not solely responsible for the formation of SAHF. To understand these differences, we mapped the difference in distal interactions between growing cells and HGPS cells (Figure 6C). Interestingly, we do not observe a spatial clustering of constitutive heterochromatin in HGPS cells, suggesting that SAHF formation is a two-step process, with the initial L1-LAD decompaction shared between cellular senescence and HGPS.

The similar trends in OCI between senescence and progeria may suggest that the perturbation of the lamina has comparable consequences, independent of its cause, and that these consequences connect the nuclear changes seen in both cell types.

A role for changes in nuclear lamina interactions and RT dynamics has been described in differentiation (Hiratani et al., 2004; Peric-Hupkes et al., 2010). Although senescence and differentiation are both being studied thoroughly, there are few studies focusing on the crosstalk between the two. Senescence has been identified as a barrier to dedifferentiation with induced pluripotent stem cell (iPSC) reprogramming (Banito et al., 2009; Li et al., 2009), and a role for senescence in embryonic development has been highlighted in two recent studies (Muñoz-Espín et al., 2013; Storer et al., 2013). The emerging concept of epigenetic rejuvenation aims to reverse the aging process without dedifferentiating target cells and depends on our ability to discriminate these two processes (Manukyan and Singh, 2012; Rando and Chang, 2012). To position the higher-order chromatin structure dynamics described above within the wider context of differentiation, we compared our Hi-C data to an ESC Hi-C data set (Dixon et al., 2012). We plotted the changes in OCI for ESCs, growing, and senescent cells over LADs, grouped by LAD overlap and isochore (Figure 6D). Remarkably, we find LAD OCI behavior in senescence to be inversed with respect to the ESC configuration (Figure 6D). L1-LADs show a similar profile in both ESCs and growing cells with an inverted profile in senescence, again highlighting the unique behavior of the L1-LADs for the senescence phenotype. The growing OCI shows an intermediate behavior between ESCs and senescent cells, suggesting a potentially continuous process of higher-order chromatin structure changes from pluripotency to senescence.

To get a more detailed impression of the dynamics of these changes, we called OCI domains in ESCs using a hidden Markov model and tested the conservation of these domains in growing and senescence cells (Figure 6E). We see a dramatic loss of the original OCI domain structure in senescence and an intermediate state in growing cells. These data suggest a close relationship between architectural events in differentiation and senescence.

## Discussion

Due to the profound changes seen within DNA-stained SAHF-positive senescent cells, a number of studies have attempted to capture the key drivers of senescence using epigenomic and microscopic techniques (Chandra et al., 2012; Cruickshanks et al., 2013; Sadaie et al., 2013; Shah et al., 2013). Using Hi-C, we have been able to generate a comprehensive description of spatial changes within senescent nuclei on a global scale.

SAHF were originally described as a gene-silencing compartment, and cellular senescence has since been associated with an increase in constitutive heterochromatin (cHC) (Narita et al., 2003; Reimann et al., 2010). Contrary to these expectations, we find a dramatic loss of local interactions in the cHC compartment, as described by an increase in OCI. Although this observation challenges the current view of SAHF, it resolves the previously paradoxical relationship between senescence and aging by correlating both with the loss of heterochromatin observed in premature and healthy aging (Scaffidi and Misteli, 2006). A loss of heterochromatin in senescence is also supported by a recent study showing the activation of satellite repeats early in cellular senescence, termed senescence-associated distension of satellites (SADS) (Swanson et al., 2013). If loss of cHC unifies senescent and aging cells, what is unique to senescence that enables SAHF formation?

Further to the general loss of heterochromatin, we show a spatial clustering of decondensing regions in cellular senescence, but not in Hutchinson-Gilford progeria syndrome (HGPS) cells. These interactions may represent a unique consecutive step required for SAHF formation, leading to a two-step mechanism where heterochromatin decondensation is followed by spatial clustering (Figure S5). A two-step mechanism for SAHF formation has also been proposed in the recent study uncovering SADS, in agreement with our findings (Swanson et al., 2013).

Whereas we can only speculate about the reason behind the L-LADs decompaction, one possible mechanism is the recently described activation of long interspersed nuclear elements involved in replicative senescence (De Cecco et al., 2013). This activation and retrotransposition was implicated in reinforcing the senescence phenotype through DNA damage.

Whereas a higher OCI could suggest an activation of the cHC compartment within senescence, a deeper investigation into the relationship with transcriptional changes will be necessary to better understand SAHF function or to break with the concept of SAHF as a silencing compartment. For example, it may be that the retention of repressive chromatin marks such as H3K9me3 and an increase in HP1 proteins is sufficient for gene silencing, despite chromatin decondensation.

Our data suggest common chromatin changes between senescence and progeria; however, we can only speculate about the upstream mechanisms leading to these similar nuclear phenotypes. Some possible crosstalks have been described in the literature, such as the activation of p53 and pRb due to progerin overexpression via a telomeric DNA damage response (Benson et al., 2010). Likewise, telomere erosion in replicative senescence was shown to induce progerin expression in normal fibroblasts (Cao et al., 2011). However, the same study found no progerin induction in oncogene-induced (telomere-independent) senescence (Cao et al., 2011). Our study establishes a link between oncogene-induced senescence and HGPS in the loss of local contacts of GC-poor LADs, suggesting a common destabilization of the nuclear lamina phenocopied by the loss of LMNB1 in senescence and the expression of progerin in HGPS.

In addition to the dramatic changes we see in L-LADs, we consistently find a GC-rich compartment of the genome gaining internal contacts in senescence. This compartment is enriched in H2 and H3 isochores and overlaps those TADs gaining boundary insulation. When compared to LMNB1 ChIP-seq data, these regions overlap with regions gaining LMNB1 in senescence (Sadaie et al., 2013). Based on our FISH data, they may locate to the nuclear periphery. It will be interesting to characterize these senescence-acquired LADs further, especially to see whether they are a senescence-specific feature or whether they exist in other cellular states.

The global extent to which regions switch their OCI in senescence is reminiscent of an inversion of the global interaction pattern. This global response starts to take effect after a few hours of c-raf induction and affects the whole population of cells after 48 hr. SAHF formation has been shown to occur downstream of several different cellular stresses. The extent of the interactome change, the response time, and the variety of triggers leads us to speculate that this nuclear restructuring may be a fundamental hardwired response of the cell. Our data show a strong correlation between OCI change and isochores; isochores may have physical properties beyond simple sequence recognition that allow the genome to rearrange its architecture during a stress response. A relationship between isochore structure and stress response would have important implications for evolution and could go some way to explaining the differences between the integration and fixation rates seen for some repetitive elements (Costantini et al., 2012).

Senescent cells share similarities with terminally differentiated cells, such as those having permanently exited the cell cycle. Furthermore, effectors of senescence like p53 and p16 have been shown to critically regulate self-renewal in adult stem cells (Janzen et al., 2006). However, there have been few studies on the relationship between senescence and differentiation. The emerging concept of epigenetic rejuvenation aims to reverse the aging process without dedifferentiating target cells (as opposed to iPSC reprogramming), thereby avoiding risks associated with pluripotency, such as cancer. Any realization of epigenetic rejuvenation depends on our ability to discriminate differentiation and senescence (Manukyan and Singh, 2012). ESCs and senescent cells show globally inverted domains of local compaction whereas somatic cells show an intermediate state between ESCs and senescence. Our observations suggest that the remodelling of the higher-order structure we describe for senescence is a continuation of ESC to somatic differentiation. Based on this preliminary observation, reversing these architectural changes may reboot the nuclear architecture in an undifferentiated ESC-like state, contrary to the concept of rejuvenation. However, a deeper understanding of distinct architectural features, distinguishing early differentiation and senescence, could provide a rationale for rejuvenation approaches.

## Experimental Procedures

### Hi-C

Hi-C was performed essentially as described in Lieberman-Aiden et al. (2009), with some modifications described in the Supplemental Experimental Procedures.

### FISH

FISH labeling was performed as described in Bolland et al. (2013). FISH probes were ordered prelabeled from empire genomics. A list of clones can be found in the Supplemental Experimental Procedures. FISH data were analyzed from confocal sections using Volocity software. Top and bottom focal planes were discarded, and the analysis was restricted to central focal planes showing a clear FISH signal. Volocity software was used for automated object detection and distance measurements.

### Cell Culture

WI-38hTERT/GFP-RAF1-ER was a generous gift from Carl Mann. Cells were cultured with 10% fetal bovine serum under 5% O2 and handled as described in Jeanblanc et al. (2012). Senescent cells were harvested 48 hr after induction with 4-OH tamoxifen.

### Cell Proliferation Assays

BrdU (anti-BrdU; PharMingen) and DAPI staining were carried out as described in Narita et al. (2003).

### Computational Data Analysis

Please refer to the Supplemental Experimental Procedures for details about the data analysis.

## Author Contributions

T.C., P.F., and W.R. designed the study. T.C., S.S., M.F.-M., J.-Y.T., and K.K. performed experiments. T.C., P.A.E., S.W.W., and S.A. analyzed the data. T.C., P.A.E., and W.R. wrote the manuscript.

## Accession Numbers

The ENA accession number for the sequencing data reported in this paper is PRJEB8073.

## Supplemental Information

Document S1. Supplemental Experimental Procedures and Figures S1–S5

Document S2. Article plus Supplemental Information
