# Supplementary material for: Global Reorganization of the Nuclear Landscape in Senescent Cells
Source: Cell Rep. 2015 Jan 29;10(4):471–83. doi: 10.1016/j.celrep.2014.12.055 (PMC4542308; doi:10.1016/j.celrep.2014.12.055)
Supplement: Document S1. Supplemental Experimental Procedures and Figures S1–S5 [file mmc1.pdf]

Cell Reports

Supplemental Information

Global Reorganization of the Nuclear Landscape in Senescent Cells

Tamir Chandra, Philip Andrew Ewels, Stefan Schoenfelder, Mayra Furlan-Magaril, Steven William Wingett, Kristina Kirschner, Jean-Yves Thuret, Simon Andrews, Peter Fraser, and Wolf Reik

Extended experimental procedures

Hi-C

Cells were fixed in 2% formaldehyde for 10 minutes. After digestion with HindIII (NEB) overnight at 37˚C, restriction sites were filled in with Klenow (NEB) using biotin-14-dATP (Life Technologies). Ligation (4 hours at 16˚C) was followed by reversal of crosslinks (65˚C overnight in the presence of Proteinase K), RNase A treatment, and phenol chloroform extraction of ligation products. The concentration DNA was determined using Quant-iT Pico Green (Life Technologies), and 25 µg of Hi-C library DNA were subjected to removal of biotin from non-ligated fragment ends, followed by sonication (Covaris E220). Sonicated DNA was end-repaired and size selected using AMPure XP beads (Beckman Coulter), before dATP-tailing with Klenow exo- (NEB). Biotin-marked ligation products were isolated using MyOne Streptavidin C1 Dynabeads (Life Technologies), and Tru-seq adapters (Illumina) were ligated onto Hi-C ligation products bound to streptavidin beads. Bead-bound Hi-C DNA was amplified with rounds of 9 PCR amplification. Hi-C libraries were interrogated by paired-end sequencing.

FISH clones:

RP11-937K23 chr18:63788654-64008278
RP11-104N14 chr18:44546277-44745145
RP11-953M14 chr9:24147300-24351523

Chromosomal interaction heatmaps

Heatmaps were generated using HOMER as described in documentation (Heinz et al., 2010). Results were viewed using Java Tree View (Saldanha, 2004).

WashU Interaction Plots

Interaction BED files were generated using WashU_Pipe and viewed on the WashU Epigenome Browser (Zhou et al., 2013). WashU_Pipe is a tool to convert BAM/SAM files generated by HiCUP into interaction BED files in a format that can be interpreted by the WashU Epigenome Browser. The absolute number of interactions between two bins determines the strength of a given interaction. WashU_Pipe can create data subsets so that only interactions between a region of interest and the remainder of the genome are displayed. Interaction counts were binned into 40kb windows and thresholds were set according to overall read counts from the libraries to normalise for sequencing depth.

TAD internal / external interactions

“Read count” and “HiC read count” quantitations were used in SeqMonk (http://www.bioinformatics.babraham.ac.uk/projects/seqmonk/), using the selected TAD as a single probe. HiC read count ignores second read pair count if within the same window, so difference gives TAD-internal interactions. “4C other ends” data import was used to gather all ditag other ends from selected TAD. Read counts were generated for the remaining parts of the chromosome and all trans chromosomes. All values were normalised for total HiC read counts between samples.

Calculation of OCI

OCI values were calculated using SeqMonk. Rolling windows were created across the genome (200kb unless otherwise stated). Raw HiC ditag counts were calculated for each window; those with no counts or extremely high counts were removed. The “cis/trans” quantitation method was then used on samples. The chromosomal median values were subtracted for all windows. The “smoothing subtraction quantitation” using a window size of 20 megabases was used to normalise for biases along the lengths of the chromosomes.
For *cis*-only OCI, *trans* reads were discarded and the “use distal reads as trans” option was used in the “cis/trans” SeqMonk quantitation, taking reads >= 20mb away as distal.

DNase hypersensitivity scatter plot
DNase hypersensitivity data was downloaded from the UCSC web browser for IMR90 cells. This was trimmed and aligned using bowtie. Reads were imported into SeqMonk and counted in the same windows used for OCI calculation. Reads counts were exported before being log transformed in R. Data was thresholded to remove extreme values and used to colour scatter plot points.

%GC Content scatter plot
The %GC content of genomic windows used for OCI scatter plot was calculated using a custom script. Data was thresholded to remove extreme values and used to colour scatter plot points.

Isochores
Isochore co-ordinates were taken from (Costantini et al., 2006). LAD – isochores were used based on their overlaps. If a LAD spans two isochores it is split, with the exception of Fig S2 where LADs were classified into isochores based on the greatest overlap.

TAD cross-boundary ratios
Counts of ditags within TADs and spanning TAD boundaries were calculated using SeqMonk with the “read count” and “HiC read count” method described above. TADs were assigned to isochores and plotted in R.

Identification of Opening and Closing TADs
Variation of the insulation of all TADs was calculated by assuming the true mean to be 0 (no difference between growing and senescence). Mean and difference between insulation scores were calculated for each point. Data was ordered by means and the standard deviation was calculated for each point using the 50 points ahead and behind it. Assuming the mean to be 0 and the data to have a normal distribution, p values were calculated using the pnorm function.

Opening TADs were called by selecting only points with $p < 0.05$. As this cutoff did not select any closing TADs, a second cutoff ($p < 0.25$) was used and filtered for only closing TADs for comparison.

Feature enrichment
Expected overlap per bp was calculated for each feature type (total length of feature / genome length). Observed scores were calculated for the overlap of each feature (overlap between region of interest and feature / total length of region of interest). Enrichment scores were calculated with $\log_2$ of (observed / expected).

Change in OCI
Change in OCI was calculated by subtracting senescence OCI scores from growing for each genomic window.

Change in interaction strengths
Interaction counts were calculated using the “4C other ends” feature in SeqMonk . Ditag ends were taken from all regions marked as a feature of interest. Other ends were ignored if falling within the same feature bin as their partner or if their partner was within 2 megabases. Overlap of resulting other ends was calculated with simple read count quantitation. Values were normalised according to total HiC read counts.

Changes in interaction were calculated by subtracting the growing scores from senescence.

Change in replication timing
Replication timing data was downloaded from http://www.replicationdomain.com/ (ChipID: 369858-3 IMR90 and 508590-2A03 IMR90 Ras). Co-ordinates were converted to hg19 genome build using the niceLiftOver tool (https://github.com/ewels/niceLiftOver). Data was binned into 200kb windows and plotted in R. Original smoothed data (unbinned) was used for the browser shot.

Hierarchical clustering
OCI was calculated using 1 megabase windows due to poor quality of progeria HiC data. Hierarchical clustering was performed using SeqMonk. %GC content of probes was calculated using a custom script and plotted using R.

Conservation of ES OCI-domains
OCI data was discretised in 200kb windows as distal or local according to whether it had a positive or negative score. OCI domains were called using a hidden markov model in R. The percentage of bins that were distal within each domain was called for the discretised ES, growing and senescent data. ES data follows a bimodal distribution of being nearly all either distal or local, as expected.

References

Costantini, M., Clay, O., Auletta, F., and Bernardi, G. (2006). An isochore map of human chromosomes. Genome Res. *16*, 536–541.

Heinz, S., Benner, C., Spann, N., Bertolino, E., Lin, Y.C., Laslo, P., Cheng, J.X., Murre, C., Singh, H., and Glass, C.K. (2010). Simple combinations of lineage-determining transcription factors prime cis-regulatory elements required for macrophage and B cell identities. Mol. Cell *38*, 576–589.

Saldanha, A.J. (2004). Java Treeview--extensible visualization of microarray data. Bioinformatics *20*, 3246–3248.

Zhou, X., Lowdon, R.F., Li, D., Lawson, H.A., Madden, P.A.F., Costello, J.F., and Wang, T. (2013). Exploring long-range genome interactions using the WashU Epigenome Browser. Nat. Methods *10*, 375–376.

Figure S1 (related to Fig 1)

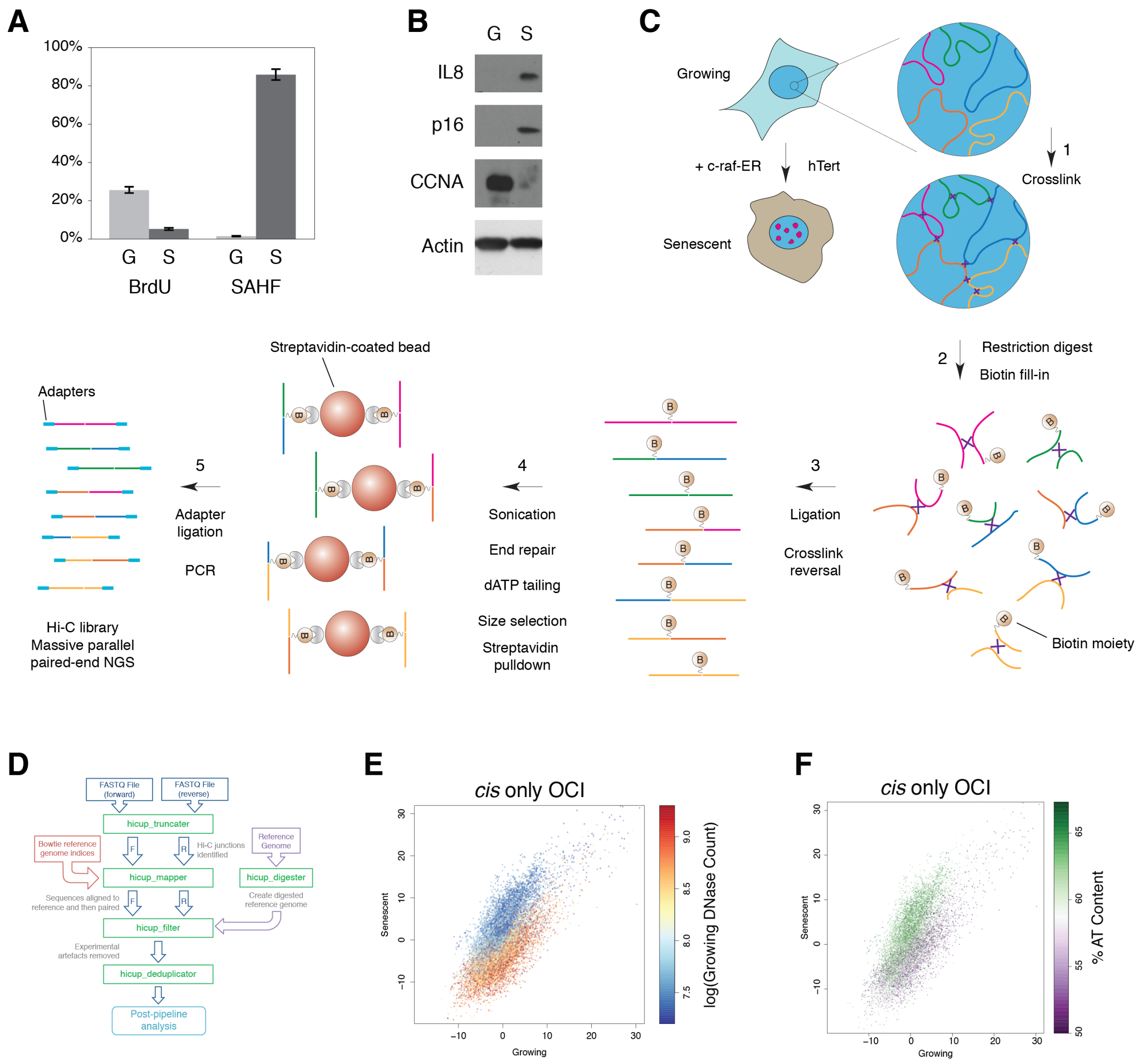

Senescence is accompanied by local and global changes in the interaction pattern of the genome (related to Figure 1).

(A) BrdU and SAHF count for growing (G) and senescent cells (S). (**B**) Western blot of key senescent markers between growing (G) and senescence (S) from whole cell lysate. **(C)** Diagram of Hi-C experimental work-flow. Cells were fixed in formaldehyde to preserve the nuclear architecture. Chromatin was digested with HindIII and restriction sites were filled in using biotinylated dATPs. The following ligation captured spatially proximal ends. Biotin-marked ligation products were enriched, amplified and sequenced. **(D)** Flow diagram showing HiCUP HiC processing data flow. **(E)** Scatter plot for OCI values calculated in cis. DNase accessibility is highlighted according to colour scale. **(F)** Scatter plot for OCI values calculated in cis. AT content is highlighted according to colour scale.

Figure S2 (related to Figs 2 and 3)

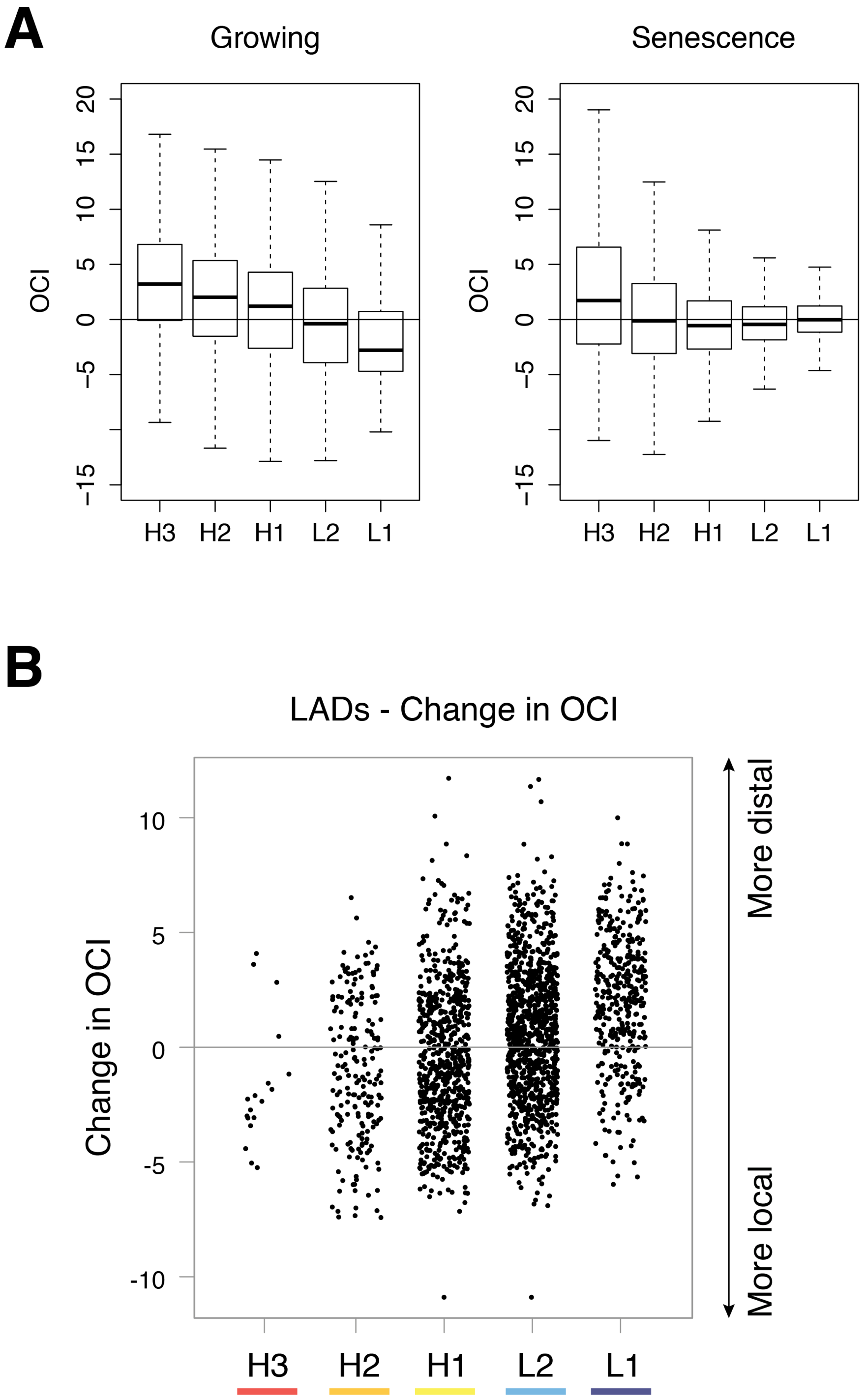

Sequence composition predicts structural chromatin dynamics in senescence (related to Figures 2 and 3)

(A) Boxplots showing distribution of OCI across different isochores in Growing and Senescent cells. **(B)** Strip-chart showing change in OCI in LADs. Each point is the average for an individual LAD.

Figure S3 (related to Fig 4)

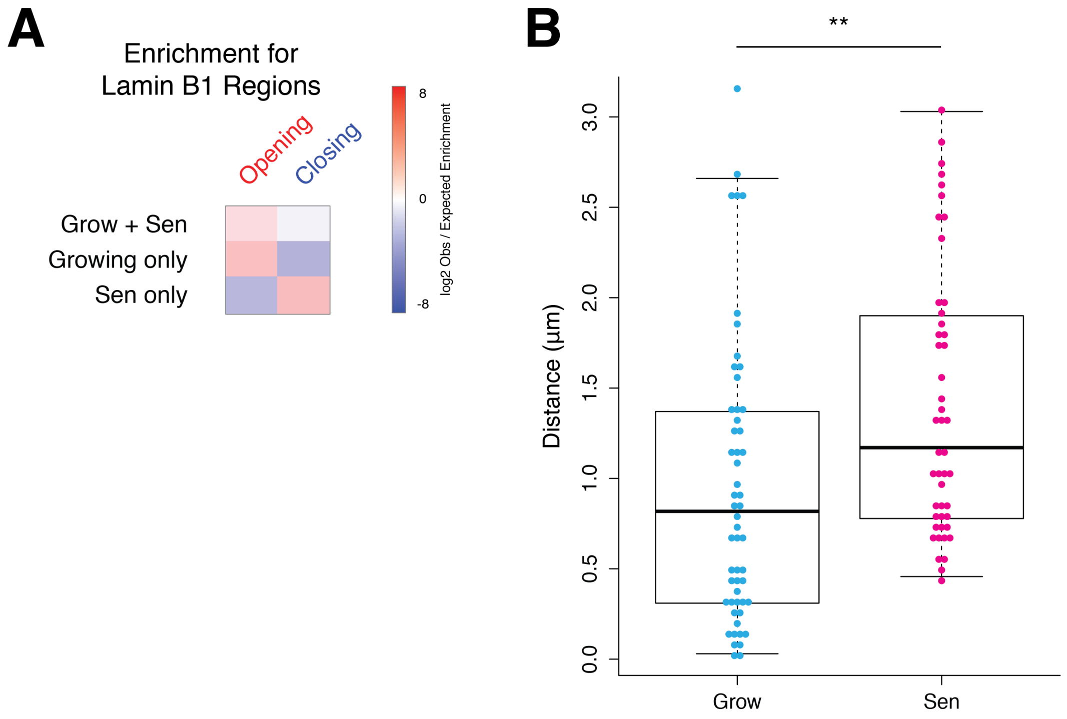

Combined sequence composition and Lamin association predict the strongest OCI gaining regions, which are changing their nuclear positioning (related to Figure 4).

(A) Enrichment of opening and closing TADs for LMNB1 regions found in growing and senescence (Grow + Sen), only in growing (Growing only) or only in senescence (Sen only). **(B)** Distance to periphery for a L1 LAD in Growing and Senescence ($p < 0.002$; Wilcoxon Mann Whitney test). Probe: RP11-953M14, chr9:24147300-24351523

Figure S4 (related to Fig 6)

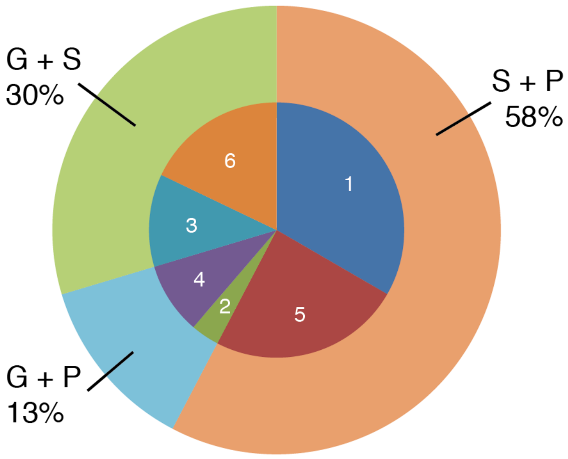

Higher order chromatin dynamics in senescence reflect changes in progeria and may represent the end point of a continuous remodelling process in differentiation (related to Figure 6).

Genome percentages of clusters from hierarchical clustering from Figure 6. The inner circle shows the genome percentage of individual clusters. The outer circle shows where growing and senescence (G+S), senescence and progeria (S+P) and growing and progeria (G+P) show similar trends in OCI.

Figure S5 (related to Fig 6 and Discussion)

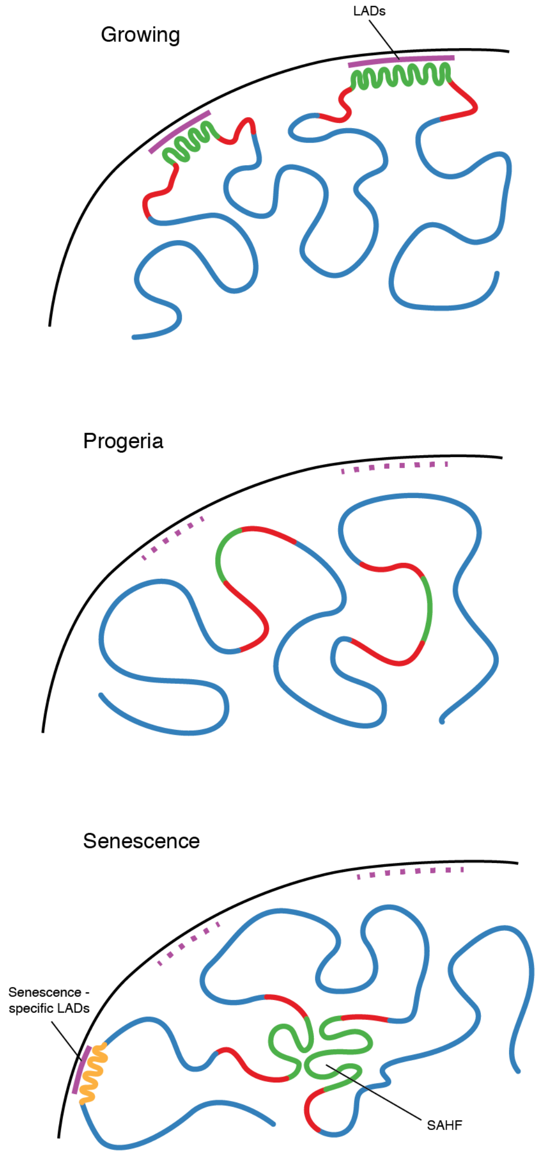

A model reconciling observations from cellular senescence and progeria (related to Figure 6 and Discussion).

Model comparing SAHF formation and HGPS. The destabilisation of LAD heterochromatic areas is common to SAHF formation and HGPS cells. SAHF formation shows a specific clustering of these heterochromatic domains.
