# Supplementary material for: Global Reorganization of the Nuclear Landscape in Senescent Cells
Source: Cell Rep. 2015 Jan 29;10(4):471–83. doi: 10.1016/j.celrep.2014.12.055 (PMC4542308; doi:10.1016/j.celrep.2014.12.055)
Supplement: Document S2. Article plus Supplemental Information [file mmc2.pdf]

Cell Reports

Article

Global Reorganization of the Nuclear Landscape in Senescent Cells

Graphical Abstract

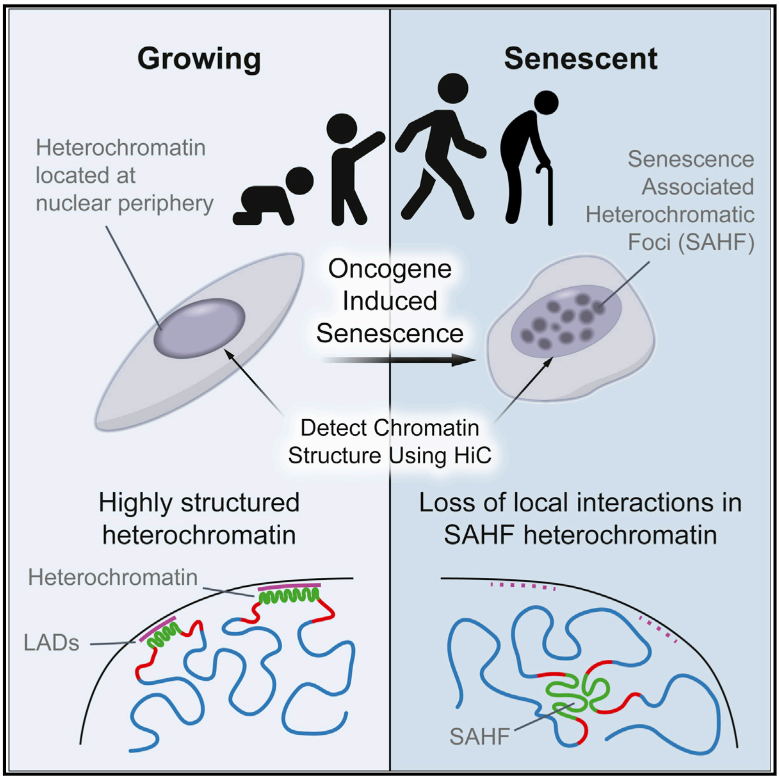

Highlights

- SAHF cells show sequence- and LAD-dependent loss of heterochromatin (HC) structure
- Senescence HC behavior is mirrored in Hutchinson-Gilford progeria
- Senescence-specific spatial clustering of HC leads to a new model for SAHF formation
- Comparing ESCs, somatic, and senescent cells links senescence to differentiation

Authors

Tamir Chandra, Philip Andrew Ewels, ..., Peter Fraser, Wolf Reik

Correspondence

tamir.chandra@babraham.ac.uk

In Brief

Chandra, Ewels, et al. map changes in genome organization in cellular senescence using Hi-C. Contrary to the believed increase in heterochromatin in senescence-associated heterochromatic foci formation, they describe a loss of local interactions in heterochromatic regions. This is in agreement with changes observed in progeria cells.

Chandra et al., 2015, Cell Reports *10*, 471–483
February 3, 2015 ©2015 The Authors
http://dx.doi.org/10.1016/j.celrep.2014.12.055

Cell Reports
Article

OPEN ACCESS
CellPress

Global Reorganization of the Nuclear Landscape in Senescent Cells

Tamir Chandra,[1,2,7,*] Philip Andrew Ewels,[1,7,8] Stefan Schoenfelder,[3] Mayra Furlan-Magaril,[3] Steven William Wingett,[3] Kristina Kirschner,[4] Jean-Yves Thuret,[5] Simon Andrews,[6] Peter Fraser,[3] and Wolf Reik[1,2]
[1]Epigenetics Programme, The Babraham Institute, Cambridge CB22 3AT, UK
[2]The Wellcome Trust Sanger Institute, Cambridge CB10 1SA, UK
[3]Nuclear Dynamics Programme, The Babraham Institute, Cambridge CB22 3AT, UK
[4]Cambridge Institute for Medical Research, University of Cambridge, Cambridge CB2 0XY, UK
[5]CEA, iBiTec-S, SBIGeM/CNRS FRE3377 I2BC/Université Paris-Sud, Gif-sur-Yvette 91191, France
[6]Bioinformatics Group, The Babraham Institute, Cambridge CB22 3AT, UK
[7]Co-first author
[8]Present address: Department of Biochemistry and Biophysics, Science for Life Laboratory, Stockholm University, Stockholm 106 91, Sweden
*Correspondence: tamir.chandra@babraham.ac.uk
http://dx.doi.org/10.1016/j.celrep.2014.12.055
This is an open access article under the CC BY license (http://creativecommons.org/licenses/by/3.0/).

SUMMARY

Cellular senescence has been implicated in tumor suppression, development, and aging and is accompanied by large-scale chromatin rearrangements, forming senescence-associated heterochromatic foci (SAHF). However, how the chromatin is reorganized during SAHF formation is poorly understood. Furthermore, heterochromatin formation in senescence appears to contrast with loss of heterochromatin in Hutchinson-Gilford progeria. We mapped architectural changes in genome organization in cellular senescence using Hi-C. Unexpectedly, we find a dramatic sequence- and lamin-dependent loss of local interactions in heterochromatin. This change in local connectivity resolves the paradox of opposing chromatin changes in senescence and progeria. In addition, we observe a senescence-specific spatial clustering of heterochromatic regions, suggesting a unique second step required for SAHF formation. Comparison of embryonic stem cells (ESCs), somatic cells, and senescent cells shows a unidirectional loss in local chromatin connectivity, suggesting that senescence is an endpoint of the continuous nuclear remodelling process during differentiation.

INTRODUCTION

Cellular senescence is an irreversible cell-cycle arrest, originally described for primary cells after long-term cell culture and attributed to telomere attrition (Hayflick and Moorhead, 1961). More recently, cellular senescence has been established as a cellular response to a variety of stresses such as DNA double-strand breaks or oncogene activation (Di Leonardo et al., 1994; Lin et al., 1998; Serrano et al., 1997).

Oncogene-induced senescence (OIS) is an intrinsic tumor suppressor mechanism, involving activation of the key tumor suppressor pathways p53 and pRB/p16INK4a. Inactivation of one or both of these pathways is found in the majority of cancers. Markers of senescence, such as p16 upregulation, are particularly prevalent in benign lesions and are often lost upon malignancy (Braig et al., 2005; Haugstetter et al., 2010; Michaloglou et al., 2005). Reactivation of p53 in mouse models of liver cancer leads to senescence with subsequent immune clearance of cancer cells (Xue et al., 2007). A key aspect of the senescence response implicated in the immune clearance is the senescence-associated secretory phenotype (SASP) (Acosta et al., 2008; Coppé et al., 2008; Kuilman et al., 2008). SASP is characterized through the secretion of cytokines, which are able to induce paracrine senescence in neighboring cells (Acosta et al., 2013). Recent work has implicated cellular senescence in normal developmental processes (Muñoz-Espín et al., 2013; Storer et al., 2013).

In addition to its role in oncogenesis, a role for senescence in organismal aging has recently been substantiated; the depletion of senescent cells has been shown to relieve symptoms in mouse models of age-related diseases, suggesting that cellular senescence may be a useful model system for organismal aging (Baker et al., 2011; López-Otín et al., 2013).

Previous work has shown that cellular senescence in human diploid fibroblasts is accompanied by a large-scale spatial rearrangement of chromatin, forming nuclear structures known as senescence-associated heterochromatic foci (SAHF). SAHF are enriched in constitutive heterochromatic markers, such as H3K9me3 and HP1 proteins (Narita et al., 2003). However, SAHF formation does not occur in all senescent cells. The proportion of cells exhibiting SAHF depends on the method of senescence induction, ranging from a few percent in replicative senescence to nearly 90% in c-raf OIS (Jeanblanc et al., 2012). In contrast, other cellular models of organismal aging such as cells

from Hutchinson-Gilford progeria syndrome (HGPS) patients show a decrease in heterochromatin and are devoid of SAHF (Scaffidi and Misteli, 2006; Shumaker et al., 2006). Cellular models of HGPS and cellular senescence of fibroblasts have proven to be relevant models for organismal aging. It is therefore important to understand the seemingly contradictory roles of heterochromatin in cellular aging and SAHF formation.

We have recently shown that SAHF chromosomes show an inversion of euchromatin, facultative heterochromatin (fHC), and constitutive heterochromatin (cHC), with cHC moving to the center of chromosomal territories (see also Figure 4C; Chandra et al., 2012). This inversion is due to a physical reorientation of the chromatin rather than a redistribution of repressive histone marks, questioning a causal role for classical heterochromatic marks, H3K9me3 (cHC) and H3K27me3 (fHC), in the formation of heterochromatin in somatic cells. A key feature of the senescent nucleus and strong correlate with SAHF formation is the loss of lamin B1 (Sadaie et al., 2013; Shah et al., 2013). Other factors involved in SAHF formation, such as the cell-cycle regulator pRb, high mobility group proteins HMGA1/HMGA2, histone chaperones HIRA and ASF1a, canonical Wnt signaling, chromatin remodelling proteins p400 and BRG1, and linker histone H1, have been identified; however, knowledge of how the chromosome structure is changed is still lacking (Chan et al., 2005; Chicas et al., 2010; Funayama et al., 2006; Narita et al., 2003, 2006; Tu et al., 2013; Ye et al., 2007a, b; Zhang et al., 2005). More importantly, the function of SAHF is controversial. Whereas the role of SAHF was initially reported as being tumor and cell-cycle suppressive (Narita et al., 2003, 2006), recent work has suggested that SAHF may in fact be proproliferative (Di Micco et al., 2011).

To gain insight into the function of SAHF, we decided to unravel the physical structure of senescent chromatin in unprecedented detail, combining fluorescence in situ hybridization (FISH) with Hi-C to map the physical changes that accompany SAHF formation. We find dramatic changes in both the global interaction network and local neighborhood of genomic regions. Surprisingly, we find distinct global changes in the interactions and compaction of certain classes of lamin-associated domains, defined by continuous genomic fragments of homogenous guanine-cytosine (GC) content (isochores; Bernardi, 2012). Contrary to the current view of enhanced heterochromatinization in SAHF formation, we find a loss of internal structure in constitutive heterochromatic (cHC) regions in cellular senescence. This loss of internal structure is accompanied by spatial clustering of the cHC regions. We further show that HGPS cells behave similarly to senescent cells in their local interaction changes but do not exhibit the spatial clustering of cHC, suggesting a two-step mechanism for SAHF formation. Finally, we investigate embryonic stem cells (ESCs) and senescent cells and find a fundamentally opposing local architecture with somatic cells representing an intermediate state.

RESULTS

Senescence Causes Global Shifts in Local Chromatin Interactions

To study OIS, we used the WI-38hTERT/GFP-RAF1-ER model system (Jeanblanc et al., 2012). Senescence is induced by the activation of the GFP-RAF1-ER kinase by addition of 4-hydroxy-tamoxifen. The nuclear phenotype in these cells is highly homogenous compared to other OIS systems such as Ras-induced senescence, with a high percentage of SAHF-positive cells (~86%; Figures 1A and S1A). Analyses of bromodeoxyuridine (BrdU) incorporation and abundance of key senescence markers at 48 hr after induction confirmed an OIS phenotype (Figures S1A and S1B).

Previous work on the reorganization of chromatin in senescence has revealed a complex change in nuclear architecture; intrachromosomal heterochromatin clusters within SAHF without redistribution of repressive histone marks along the linear DNA sequence (Chandra et al., 2012; see also Figure 4C). These observations suggest a dynamic remodelling of the nucleus driven by spatial changes, as opposed to a change in chromatin identity (Chandra and Narita, 2013). To further understand these changes in nuclear architecture in growing and senescent cells, we performed Hi-C, a proximity-based ligation assay that measures the frequency of close chromatin interactions, as summarized in Figure S1C (Lieberman-Aiden et al., 2009). Two independently grown and fixed batches of cells were sequenced to a depth of at least 114 million reads. Data were processed using the HiCUP pipeline (S.W.W. and S.A., unpublished data; Figure S1D). For initial inspection, we plotted Hi-C interaction heatmaps using HOMER (Figure 1B; Heinz et al., 2010). The heatmaps show a typical pattern of self-interacting topologically associated domains (TADs) (Dixon et al., 2012; Nora et al., 2012). Although the global chromosomal interaction pattern appears largely unchanged between growing and senescent cells, closer inspection shows striking differences in the strength of internal interactions within TADs (Figures 1B and 1C). To further investigate this phenomenon, we calculated the number of interactions in which both interacting regions are located within a single TAD versus those spanning the TAD boundaries (Figure 1C). This revealed a loss of interactions within the TAD and a corresponding gain of cross-boundary interactions in senescent cells (Figure 1C). To assess the consequences of loss of local contacts for chromatin compaction, we measured the distance between two FISH probes (separated by ~2.2 Mb)—one approximately 100 kb upstream of the TAD and the corresponding probe inside the TAD (Figures 1D and 1F for probe locations). We found a substantial shift in the mean distance separating the probes from 0.57 μm in growing to 0.86 μm in senescent cells (Wilcoxon-Mann-Whitney test; $p = 0.00003$). This observation confirms for this TAD that a loss of local interactions leads to a change in physical compaction in senescence.

To study this change in local interaction frequency more precisely, we developed a measure to examine the difference in local versus distal interactions genome wide, which we call open chromatin index (OCI) (Figure 1E). This analysis counts the number of interactions with both Hi-C reads within a window and compares these to a control. Initially, we used interaction counts across the remainder of the chromosome as a control but found that results were biased due to the varying lengths of chromosomes. As an alternative, we used the number of interchromosomal contacts. The distal and interchromosomal interaction counts behaved concordantly (Figure 1C), and the latter provided a more stable metric. We measured the ratios of these

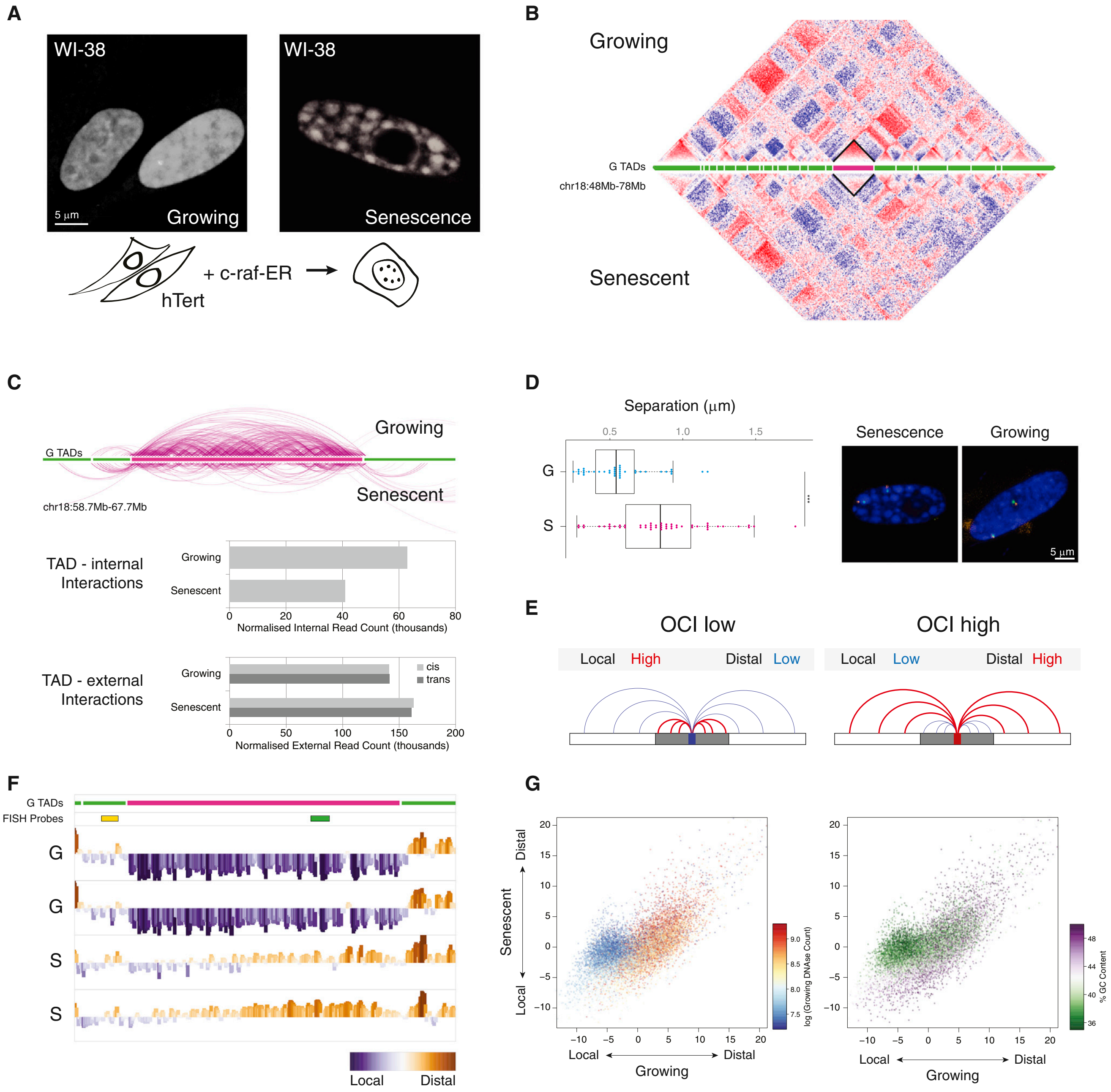

Figure 1. Senescence Is Accompanied by Local and Global Changes in the Interaction Pattern of the Genome

(A) WI-38 hTERT/GFP-RAF1-ER cells show the characteristic SAHF phenotype upon senescence induction.

(B) Interaction heatmaps for chr18q for growing (top) and senescence (bottom). Strong interactions are shown in red whereas weak interactions are in blue. Green lines (center) represent topologically associating domains (TADs) determined in growing cells. Highlighted TAD (black rhombus and pink TAD line) suggests a loss of internal interactions.

(C) Top: genome browser shot of interactions reaching into and from within the depicted TAD. Growing cells show more TAD internal interactions than senescent cells. Bottom: normalized read counts from highlighted TAD. Internal contacts confirm loss of TAD internal interactions and external interactions increase in senescence, both in *cis* (light gray) and in *trans* (dark gray).

(D) DNA-FISH showing separation between probes located within and adjacent to the highlighted TAD. Separation increases in senescence $p = 0.00003$ (Mann-Whitney-Wilcoxon test). Shown to the right are representative confocal microscopy planes of the FISH separation experiment.

(E) Top: schematic of the open chromatin index (OCI), which describes regions changing their ratio between local and distal contacts.

(F) Browser shot of OCI in two biological replicates (G, growing; S, senescence) over highlighted TAD (pink) shows a loss of local interactions.

(G) Scatterplots comparing genome-wide OCI in 200 kb windows between growing and senescence. Points are colored by DNase accessibility as measured in growing fibroblasts (left) and %GC content (right). A cluster of points can be seen to deviate from the diagonal, which shows a loss of local contacts in senescence. These regions are the least accessible in the genome and have a low GC content.

interactions in windows across the genome and normalized by subtracting the median chromosomal value from each window and then by smoothing values in a rolling 20 Mb window. The resulting OCI values give insight into the propensity of a region to form local or distal interactions. Our measurement bears some resemblance to the previously described interchromosomal contact propensity (ICP) (Kalhor et al., 2012), which itself has been found to correlate with active marks such as RNA polymerase II occupancy (Kalhor et al., 2012). When visualized over the TAD shown in Figures 1B and 1C, we find a rise in the OCI in senescence, suggesting OCI may be a suitable metric to measure a switch from local to distal interactions (Figure 1F). To explore OCI changes genome wide, we plotted OCI values in growing and senescence in a scatterplot (Figure 1G). A population of regions appears to follow the behavior of the TAD described above: an initially low OCI in growing cells with a rise in senescent cells, indicating a loss of local interactions. It has been previously suggested that high ICP values could be affected by proximity to the periphery of chromosomal territories (Kalhor et al., 2012). To rule out any such effect in our measurements, we calculated the OCI using only *cis* contacts, counting any interaction spanning more than 20 Mb as a distal contact (Figures S1E and S1F). We readily identified the same changing regions, confirming OCI as a suitable metric to measure changes in local architecture.

Based on the TAD shown in Figures 1B, 1C, and 1F, a low OCI in growing cells would suggest a compact structure with strong local interactions. To test this hypothesis, we correlated OCI with DNase accessibility in growing cells (Figure 1G). Corroborating our hypothesis, we found a striking overlap between the least-accessible regions in growing cells (dark blue) and regions showing the strongest rise in OCI. Next, we analyzed whether genomic regions with changing chromosomal interactions display a characteristic sequence-composition signature. We highlighted the GC content of each point within the ICP scatterplot and again identified a strong correlation, with those regions losing internal contacts strongly enriched for low GC content (Figure 1G).

Using OCI, we have identified regions of chromatin losing internal structure. These regions are the least accessible in the genome in growing cells and are rich in adenine-thymine (AT) content.

Sequence Composition Predicts Structural Chromatin Dynamics in Senescence

The bias in the GC content of regions showing changing OCI (Figure 1G) led us to investigate the role of sequence composition within senescent nuclear reorganization. We used a recently published annotation to split the genome into isochores, large blocks of similar GC content (in GC%: L1 <37; L2 37–41; H1 41–46; H2 46–53; and H3 >53), which show little compositional heterogeneity (Costantini et al., 2006). Isochores were originally resolved according to their behavior in density gradient centrifugation, adding a dimension of macromolecular behavior to our analysis of sequence content (Macaya et al., 1976; Thiery et al., 1976).

To see how isochores behave in growing and senescent cells, we plotted the OCI for each isochore (Figures 2A and S2A). We observe a large difference in the behavior between L-isochores (L1 and L2) and H-isochores (H1, H2, and H3), with the majority of regions losing their local interactions within the GC-poor isochores (Figure 2A). Our data extend the role of the sequence composition in higher-order chromatin dynamics in senescence to the physical entities of isochores.

TADs are self-interacting domains with boundaries defined by a change in the directionality bias of interacting fragments. We calculated the position of TAD boundaries within growing and senescent cells, as previously described (Dixon et al., 2012). We found a high 89.3% percent of the domains to be conserved between growing and senescent cells.

TAD boundary strength and decreased TAD internal contacts have been shown to be affected by acute loss of a functional cohesion complex (Sofueva et al., 2013). Interestingly, a recent report suggests a loss of CTCF at *CDKN2A* in oncogene-induced senescence (Hirosue et al., 2012). To test whether TAD boundaries are affected by a change in OCI, we calculated the ratio of interactions found within TADs versus those spanning a boundary (Figure 2B). We found a change in insulation strength that correlates with isochores. L1 TAD boundaries show a striking loss of boundary strength (“opening”), whereas H2 and H3 TAD boundaries appear to strengthen slightly (“closing”; Figures 2B and 2C). Thus, whereas the position of TAD boundaries remains largely unchanged in senescent cells, their quality is affected by changes in local and distal interactions, with a significant fraction of TADs losing insulation strength and a smaller fraction gaining insulation strength.

To test the changing TADs for enrichment in genomic features, we selected regions based on the significance of the difference in insulation strength ($p < 0.05$ for opening TADs; $p < 0.25$ for closing TADs; Figure 2C). We find opening TADs are enriched in H3K9me3, late replication timing (RT), and lamina-associated domains (LADs). TADs with stronger boundary insulation show a strong underrepresentation in these features and are instead enriched in regions of early replication and H3K36me3.

Sequence Composition and Lamin Association Predict OCI Increase in Senescence

Recent research has highlighted the importance of LADs and the loss of LMNB1 for the senescence phenotype (Sadaie et al., 2013; Shah et al., 2013; Shimi et al., 2011). The level of LMNB1 reduction was shown to predict SAHF-positive cells in a heterogeneous population, and ectopic expression of LMNB1 was able to reduce the number of SAHF-positive cells (Sadaie et al., 2013). L1 isochores showed the strongest loss of local interactions (Figure 2A) and TAD isolation strength (Figure 2B) and were strongly enriched in LADs (Figure 2C). To understand the behavior of LADs, we plotted the change in average OCI across LADs (Figure S2B). We find a strong correlation between isochore class and LAD OCI behavior, with L1 LADs losing and H-LADs gaining local interactions. These observations allow us to predict LAD behavior by sequence content alone.

To exclude the possibility that this observation is due only to the enrichment of LADs within AT-rich regions of the genome, we calculated OCI changes across LAD and inter-LAD regions of each isochore (Figure 3A). We find that the loss of internal contacts for L-isochores (a rise in OCI) is LAD dependent, with

OPEN ACCESS
CellPress

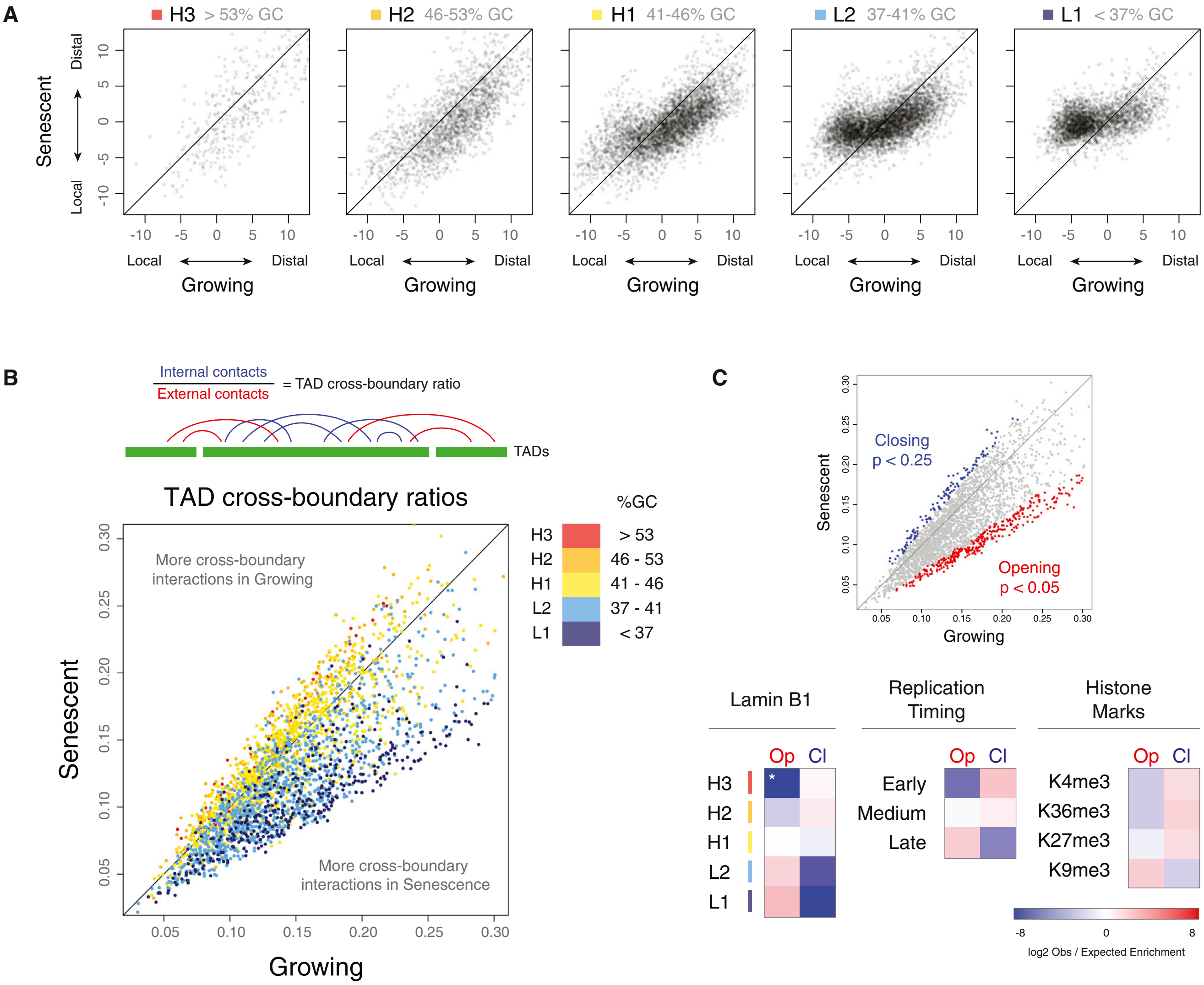

Figure 2. Sequence Composition Predicts Structural Chromatin Dynamics in Senescence

(A) Scatterplots showing OCI calculated in 200 kb windows for growing and senescence cells, separated by overlap with isochores. The greatest changes can be seen to occur in the L1 isochores.

(B) Top: schematic of TAD boundary strength calculation. Bottom: scatterplot comparing the cross-boundary ratios over TADs genome wide, colored by isochore. The L-isochores can be seen to gain cross-boundary interactions in senescence.

(C) Top: selection of opening and closing TADs highlighted in scatter plot (a less-stringent cutoff was chosen for closing TADs, in order to reach a comparable number). Bottom: enrichment for overlap with genomic features (log2 obs/exp; also see Experimental Procedures) for opening (Op) and closing (Cl) TADs.

L-inter LADs showing no change in OCI. This suggests that the combination of being in an AT-rich L-isochore and a LAD predicts the dramatic OCI gain in senescence. For H2 and H3 isochores, the OCI change is not dependent on lamin binding (Figure 3A), suggesting that other features control the behavior of these LADs.

Senescence is accompanied by a major loss of LMNB1, and recent chromatin immunoprecipitation sequencing (ChIP-seq) experiments have identified the majority of the regions losing LMNB1 occupancy in senescence (Sadaie et al., 2013). Despite this dramatic loss, a subset of regions not bound by LMNB1 in growing cells appears to gain binding in senescence (Sadaie et al., 2013). We calculated the enrichment of these growing- and senescent-specific LMNB1 regions across the isochore LADs and inter-LAD regions (Figure 3B). We observe a strong enrichment for areas losing LMNB1 in L-isochore lamin-associated domains (L-LADs), suggesting the loss of LMNB1 may be involved in the architectural changes we have uncovered. There was no enrichment of LMNB1-gaining regions in any LAD category, supporting the previous observation that these regions are unique to senescence. GC-rich inter-LADs were enriched for senescence-specific LMNB1 binding, showing the opposite isochore pattern to the loss of lamin. We tested the overlap between opening and closing TADs and

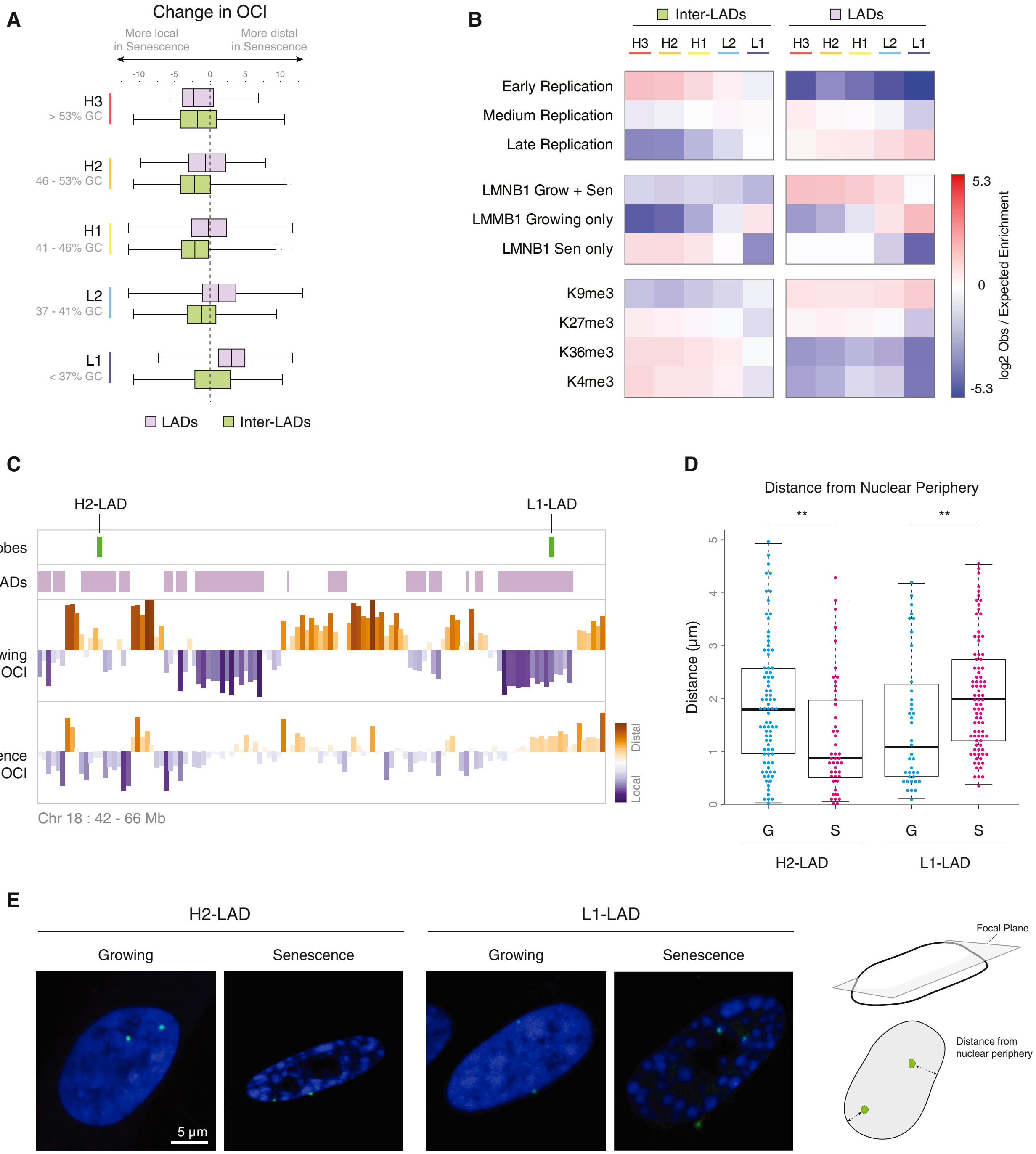

Figure 3. Combined Sequence Composition and Lamin Association Predict the Strongest OCI-Gaining Regions, which Are Changing Their Nuclear Positioning

(A) Change in OCI levels in senescence for regions overlapping LADs (purple) and inter-LADs (iLADs, green).

(B) Enrichment of LADs and iLADs for replication timing, LMNB1 regions, and several chromatin marks.

(C) Browser shot indicating genomic location for FISH probes designed against two adjacent H2-LAD and L1-LAD regions (green vertical lines).

(D) Distances of FISH signals to the nuclear periphery.

(E) Left: representative confocal microscopic images of FISH-treated growing and senescent cells. Right: schematic showing the measurements made for the DNA-FISH from the central focal plane.

growing/senescence-specific LMNB1 regions (Figure S3A). We find a strong enrichment for the newly forming senescence-specific LADs within TADs exhibiting greater insulation, suggesting a functional role for the senescence-specific gain of LMNB1. Our data suggest a role for the chromosomal redistribution of LMNB1 in the architectural changes we described.

To investigate whether the changes in LAD OCI are accompanied by changes in positioning in the nucleus, we used DNA-FISH microscopy to measure the distance of specific LADs to the nuclear periphery. We designed probes within two adjacent L1- and H2-LADs (Figure 3C); the L1-LAD showed a significant increase in the mean distance to the periphery (Figures 3D and 3E; $p < 0.006$). Interestingly, the H-LAD also showed a significant change, moving closer to the periphery (Figures 3D and 3E; $p < 0.009$). We tested another L1-LAD in close proximity to the *CDKN2A* locus and observed a similar move away from the nuclear periphery (Figure S3B; $p < 0.002$). These observations suggest that the consequences of chromatin restructuring go beyond the change in local interactions described by OCI and could additionally entail a change in nuclear positioning, with L1 LADs moving away from the periphery and H-LADs moving closer to it.

Local Changes Are Accompanied by the Formation of Specific Distal Interactions

Through changes in OCI and TAD insulation strength, we observe a redistribution of local interactions to more distal interactions for L-LADs. To test whether these new distal contacts are distributed randomly or form specific interactions, we mapped the change in normalized interaction count between different LADs. We calculated all reads linking selected features while ignoring interactions separated by less than 2 Mb to avoid any distance effects (Figure 4A). We observe a strong increase in the number of interactions between L-LADs, with the strongest gain found between L1-LADs (Figure 4B). In contrast, we do not observe any major changes in interactions between H-LADs.

Whereas L-LADs lose internal contacts, they increase their interactions between each other across the genome, indicating that they cluster together in nuclear space. This observation is reminiscent of the rearrangement of heterochromatin observed with microscopy during SAHF formation in Ras-induced senescence (Figure 4C; Chandra et al., 2012). We therefore analyzed whether interactions between domains defined by specific histone marks change in senescence. Consistent with previous microscopy data, we find that H3K9me3 domains come together in a specific manner during senescence (Figure 4D).

The concerted local loss of internal contacts and global clustering of L-LADs leads us to the model depicted in Figure 4E, whereby lamin-bound regions detach from the nuclear periphery and cluster within the nuclear interior. Some GC-rich H2/H3 regions appear to have gained internal structure and relocate to the periphery, consistent with a scenario of nuclear reshuffling resulting from LAD relocation.

Analyzing distal contacts of regions dramatically changing their local structure (L1-LADs and H3K9me3 domains) suggests a clustering of these regions within the nucleus. Previous work has speculated that SAHF may represent a special heterochromatic compartment, supported by immunofluorescence studies using DNA stains such as DAPI which show a very high intensity signal within the SAHF core. Contrary to this theory, our initial observations using Hi-C data suggest a loss of internal structure and possible decompaction of DNA enriched in the SAHF core. The clustering shown here can now explain the perceived expansion and clustering of heterochromatin and provides a new depth of understanding of the structural organization of senescent chromatin.

Pre-senescent Replication Timing Predicts Chromatin Changes

OIS shows an accumulation of cells arrested in S phase. This has been linked to replication-stress-induced DNA damage signaling, possibly caused by an initial hyperproliferative burst following oncogene activation (Bartkova et al., 2006; Di Micco et al., 2006). Although the relationship between structural chromatin changes and cellular senescence is not well understood, one key protein required for SAHF formation is pRB, linking higher-order chromatin changes and replication (Narita et al., 2003). The interplay between replication timing and SAHF architecture has also been shown by pulse-chase studies, tracking nucleoside analogue incorporation (Chandra et al., 2012).

Having identified the regions with the most dynamic structural changes in the Hi-C data, we tested whether these regions show any change in pre-senescence replication timing (RT). We reanalyzed whole-genome replication timing data from IMR90 ER-Ras cells, comparing growing cells and presenescent cells (48 hr after induction of Ras; Chandra et al., 2012). SAHF formation takes longer in ER-Ras cells, and kinetics of senescence establishment are better described, suggesting that changes observed at 48 hr would precede SAHF formation (Young et al., 2009). To compare the two data sets, we measured the change in normalized replication timing (Figure 5A). We split the genome into LADs and inter-LADs and then isochores. We see a shift toward earlier RT for L1-LADs, whereas L1 iLADs show little change. The scatterplots for the L1 isochore indicates a split between LADs (green) and iLADs (purple) into late and early replication timing, respectively (Figure 5B). A subpopulation of L1 LADs appears to be biased toward earlier RT in presenescence. An example of a locus exhibiting this shift can be seen in Figure 5C, which shows a region surrounding an L1-LAD losing its internal contact bias and changing in presenescence RT.

We find a correlation between the most significant structural changes in senescence as revealed by Hi-C and RT changes in hyperproliferating presenescent cells. This observation may indicate a role for replication timing in shaping the senescence chromatin landscape.

Higher-Order Chromatin Dynamics in Senescence Reflect Changes in Progeria and May Represent the Endpoint of a Continuous Remodelling Process in Differentiation

The role of p16-dependent senescence in organismal aging highlights the importance of cellular senescence as an in vitro aging model (Baker et al., 2011). Fibroblasts belonging to patients suffering from Hutchinson-Gilford progeria syndrome (HGPS) undergo premature senescence and also provide an important model to study aging. However, OIS and HGPS cells

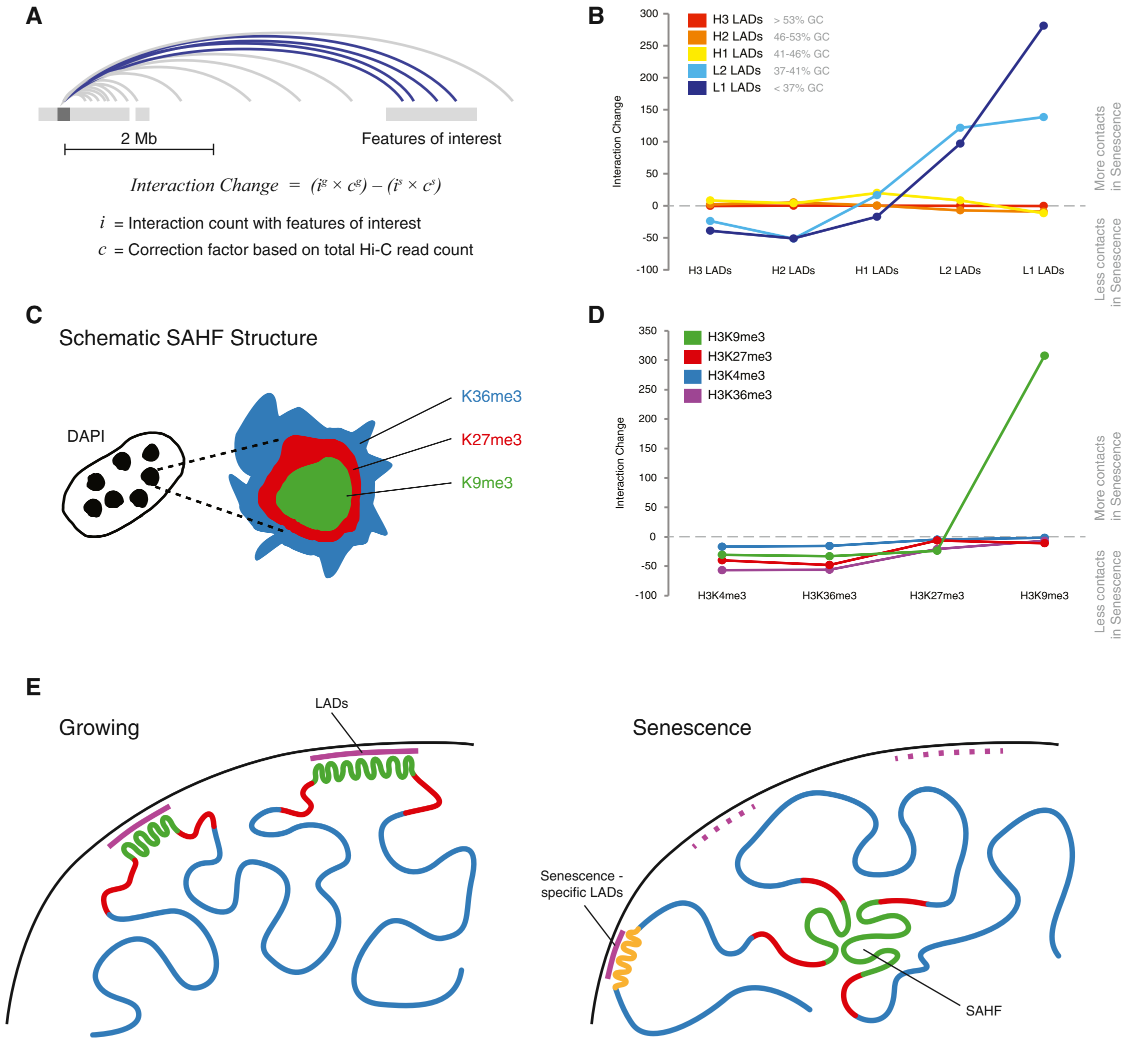

Figure 4. Local Changes Are Accompanied by the Formation of Specific Distal Interactions

(A) Schematic showing how the change in interaction strength between features was calculated. *g* and *s* supertext denotes growing and senescence. Note the avoidance of features within 2 Mb.

(B) Change in interaction strength calculated between isochore LADs comparing growing and senescence

(C) Schematic depicting the chromatin organization of histone marks in SAHF as described in Chandra et al. (2012).

(D) Change in interaction strength calculated between regions associated with histone marks.

(E) Model depicting the change of the chromatin architecture. We propose that L-LAD regions with strong local interactions (high OCI) in growing (green) detach from the nuclear lamina and lose their internal structure (potentially forming the core of the SAHF). Other regions, such as selected H-LADs, move toward the nuclear periphery.

have distinct chromatin features; for example, SAHF formation is exclusive to OIS. Whereas OIS is accompanied by a major loss in LMNB1, premature aging in HPGS is due to the accumulation of progerin, a mutated version of LMNA/LMNC (Eriksson et al., 2003). As such, a commonality found within both models is the destabilization of the nuclear lamina. To investigate the breadth of our findings regarding local chromatin changes in senescence and to examine similarities between OIS and HGPS fibroblasts, we compared our data to recently published Hi-C data sets in HGPS fibroblasts (McCord et al., 2013).

Unsupervised hierarchical clustering of OCI in growing, senescent, and progeroid fibroblasts split the data into six classes, which show strong correlation with GC content (Figure 6A). Classes 1 and 5 show similar trends for senescence and progeria and represent 58% of the genome (Figure S4). Class 1 is GC poor and highly enriched in L1-LADs (Figure 6B), suggesting a common loss of internal structure for these regions in both senescence and progeria. Cluster 5 shows the strongest enrichment of all clusters for regions gaining LMNB1 in senescence, suggesting that a common mechanism may lead to the compaction of these regions.

Whereas the decompaction of heterochromatin for HGPS cells is consistent with previous observations (Scaffidi and Misteli, 2006; Shumaker et al., 2006), the congruency in changing OCI

OPEN ACCESS
CellPress

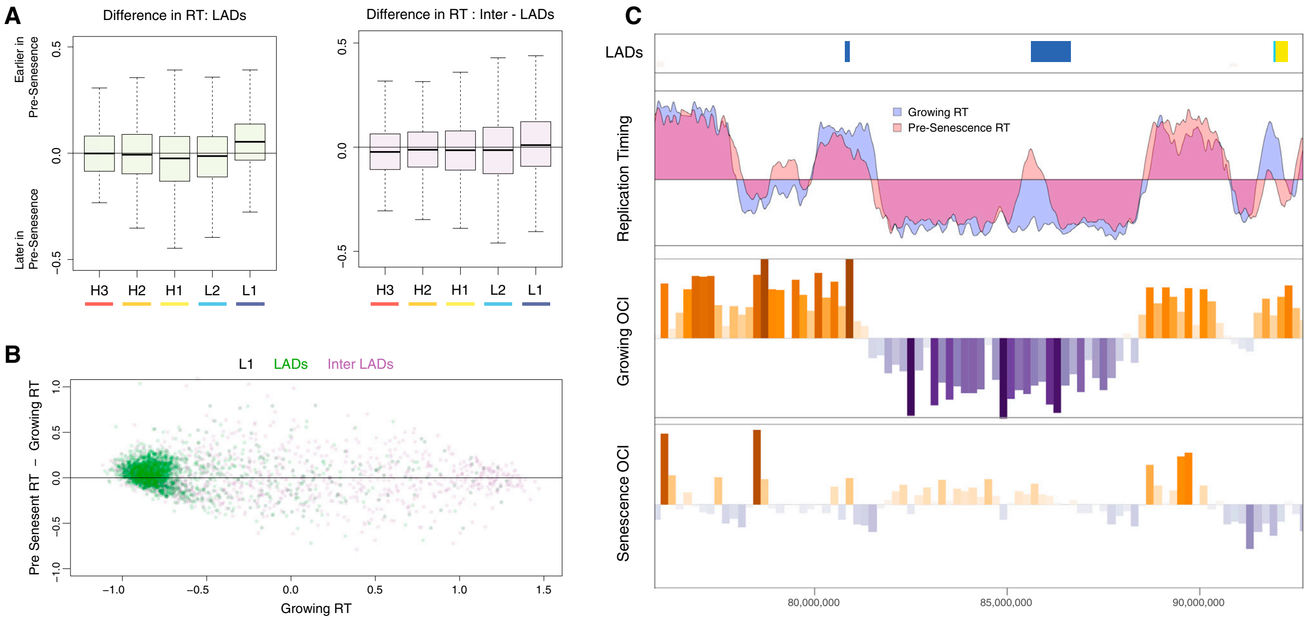

Figure 5. Presenescent Replication Timing Predicts Chromatin Changes

(A) Change in replication timing between growing OIS ER-Ras cells and presenescent cells. Regions overlapping LADs are shown in green and iLADs in purple, divided by isochore.

(B) Scatterplot showing difference between presenescent RT and growing RT versus growing RT for isochore L1; LADs (green) and iLADs (purple) are highlighted. Points above the horizontal are replicating earlier in presenescent cells.

(C) Browser shot showing a L1-LAD region changing in presenescent RT.

seems to contradict the fact that HGPS cells do not form SAHF, suggesting that changes in OCI are not solely responsible for the formation of SAHF. To understand these differences, we mapped the difference in distal interactions between growing cells and HGPS cells (Figure 6C). Interestingly, we do not observe a spatial clustering of constitutive heterochromatin in HGPS cells, suggesting that SAHF formation is a two-step process, with the initial L1-LAD decompaction shared between cellular senescence and HGPS.

The similar trends in OCI between senescence and progeria may suggest that the perturbation of the lamina has comparable consequences, independent of its cause, and that these consequences connect the nuclear changes seen in both cell types.

A role for changes in nuclear lamina interactions and RT dynamics has been described in differentiation (Hiratani et al., 2004; Peric-Hupkes et al., 2010). Although senescence and differentiation are both being studied thoroughly, there are few studies focusing on the crosstalk between the two. Senescence has been identified as a barrier to dedifferentiation with induced pluripotent stem cell (iPSC) reprogramming (Banito et al., 2009; Li et al., 2009), and a role for senescence in embryonic development has been highlighted in two recent studies (Muñoz-Espín et al., 2013; Storer et al., 2013). The emerging concept of epigenetic rejuvenation aims to reverse the aging process without dedifferentiating target cells and depends on our ability to discriminate these two processes (Manukyan and Singh, 2012; Rando and Chang, 2012). To position the higher-order chromatin structure dynamics described above within the wider context of differentiation, we compared our Hi-C data to an ESC Hi-C data set (Dixon et al., 2012). We plotted the changes in OCI for ESCs, growing, and senescent cells over LADs, grouped by LAD overlap and isochore (Figure 6D). Remarkably, we find LAD OCI behavior in senescence to be inversed with respect to the ESC configuration (Figure 6D). L1-LADs show a similar profile in both ESCs and growing cells with an inverted profile in senescence, again highlighting the unique behavior of the L1-LADs for the senescence phenotype. The growing OCI shows an intermediate behavior between ESCs and senescent cells, suggesting a potentially continuous process of higher-order chromatin structure changes from pluripotency to senescence.

To get a more detailed impression of the dynamics of these changes, we called OCI domains in ESCs using a hidden Markov model and tested the conservation of these domains in growing and senescence cells (Figure 6E). We see a dramatic loss of the original OCI domain structure in senescence and an intermediate state in growing cells. These data suggest a close relationship between architectural events in differentiation and senescence.

DISCUSSION

Due to the profound changes seen within DNA-stained SAHF-positive senescent cells, a number of studies have attempted to capture the key drivers of senescence using epigenomic and microscopic techniques (Chandra et al., 2012; Cruickshanks et al., 2013; Sadaie et al., 2013; Shah et al., 2013). Using Hi-C, we have been able to generate a comprehensive description of spatial changes within senescent nuclei on a global scale.

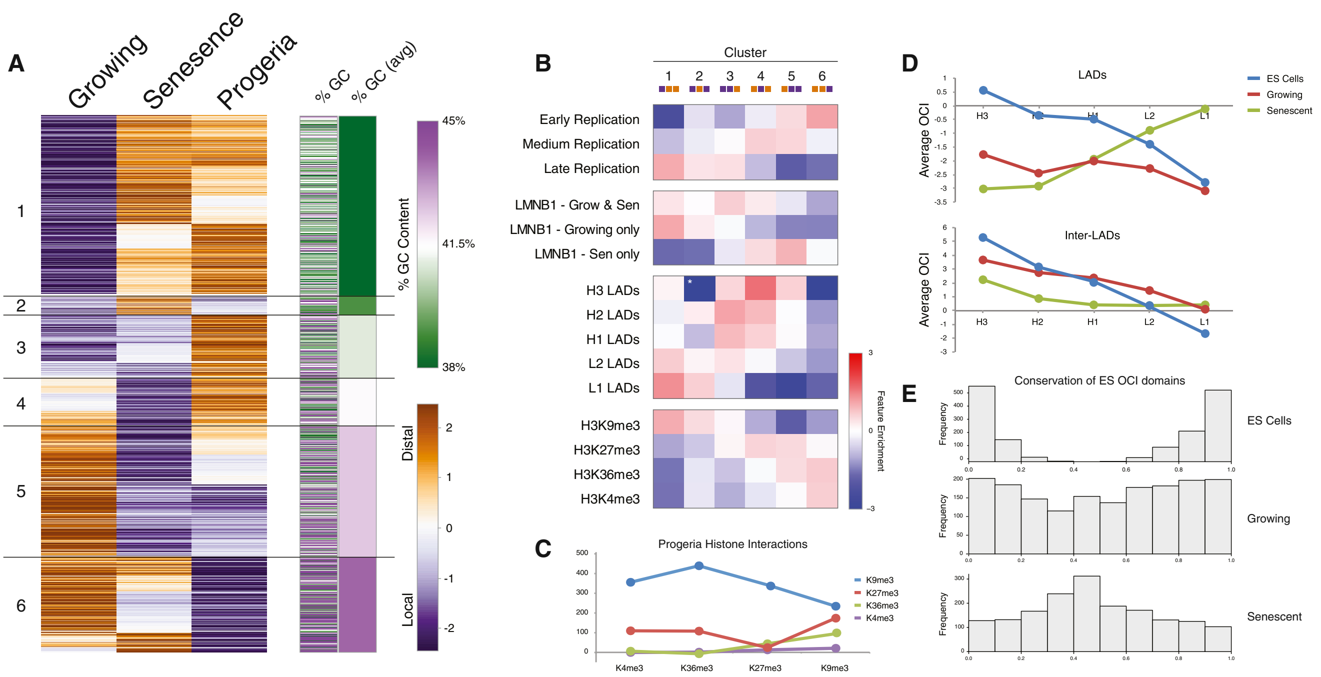

Figure 6. Higher-Order Chromatin Dynamics in Senescence Reflect Changes in Progeria and May Represent the Endpoint of a Continuous Remodelling Process in Differentiation

(A) Hierarchical clustering of OCI values in 1 Mb of genomic windows for growing, senescence, and progeria. Columns to the right show GC content per genomic window and the average %GC per cluster.

(B) Enrichment of clusters over genomic features. Note that clusters 1 and 5 show similar behavior in growing and senescence.

(C) Change of interactions calculated between regions associated with histone marks. Progeria shows no clustering of H3K9me3 regions or other histone marks compared to growing cells. Enrichment calculated as shown in Figure 4A.

(D) Average OCI over LADs (top) and iLADs (bottom) split by isochore in ESCs, growing, and senescence.

(E) Conservation of OCI domains called using a hidden Markov model (see Experimental Procedures) in ESCs. The x axis shows percentage of windows within domains classed as local (0) or distal (1). ESCs show a bimodal distribution as expected. Growing and senescent cells show a decaying conservation of interaction state within these domains.

SAHF were originally described as a gene-silencing compartment, and cellular senescence has since been associated with an increase in constitutive heterochromatin (cHC) (Narita et al., 2003; Reimann et al., 2010). Contrary to these expectations, we find a dramatic loss of local interactions in the cHC compartment, as described by an increase in OCI. Although this observation challenges the current view of SAHF, it resolves the previously paradoxical relationship between senescence and aging by correlating both with the loss of heterochromatin observed in premature and healthy aging (Scaffidi and Misteli, 2006). A loss of heterochromatin in senescence is also supported by a recent study showing the activation of satellite repeats early in cellular senescence, termed senescence-associated distension of satellites (SADS) (Swanson et al., 2013). If loss of cHC unifies senescent and aging cells, what is unique to senescence that enables SAHF formation?

Further to the general loss of heterochromatin, we show a spatial clustering of decondensing regions in cellular senescence, but not in Hutchinson-Gilford progeria syndrome (HGPS) cells. These interactions may represent a unique consecutive step required for SAHF formation, leading to a two-step mechanism where heterochromatin decondensation is followed by spatial clustering (Figure S5). A two-step mechanism for SAHF formation has also been proposed in the recent study uncovering SADS, in agreement with our findings (Swanson et al., 2013).

Whereas we can only speculate about the reason behind the L-LADs decompaction, one possible mechanism is the recently described activation of long interspersed nuclear elements involved in replicative senescence (De Cecco et al., 2013). This activation and retrotransposition was implicated in reinforcing the senescence phenotype through DNA damage.

Whereas a higher OCI could suggest an activation of the cHC compartment within senescence, a deeper investigation into the relationship with transcriptional changes will be necessary to better understand SAHF function or to break with the concept of SAHF as a silencing compartment. For example, it may be that the retention of repressive chromatin marks such as H3K9me3 and an increase in HP1 proteins is sufficient for gene silencing, despite chromatin decondensation.

Our data suggest common chromatin changes between senescence and progeria; however, we can only speculate about the upstream mechanisms leading to these similar nuclear phenotypes. Some possible crosstalks have been described in the literature, such as the activation of p53 and pRb due to progerin overexpression via a telomeric DNA damage response

(Benson et al., 2010). Likewise, telomere erosion in replicative senescence was shown to induce progerin expression in normal fibroblasts (Cao et al., 2011). However, the same study found no progerin induction in oncogene-induced (telomere-independent) senescence (Cao et al., 2011). Our study establishes a link between oncogene-induced senescence and HGPS in the loss of local contacts of GC-poor LADs, suggesting a common destabilization of the nuclear lamina phenocopied by the loss of LMNB1 in senescence and the expression of progerin in HGPS.

In addition to the dramatic changes we see in L-LADs, we consistently find a GC-rich compartment of the genome gaining internal contacts in senescence. This compartment is enriched in H2 and H3 isochores and overlaps those TADs gaining boundary insulation. When compared to LMNB1 ChIP-seq data, these regions overlap with regions gaining LMNB1 in senescence (Sadaie et al., 2013). Based on our FISH data, they may locate to the nuclear periphery. It will be interesting to characterize these senescence-acquired LADs further, especially to see whether they are a senescence-specific feature or whether they exist in other cellular states.

The global extent to which regions switch their OCI in senescence is reminiscent of an inversion of the global interaction pattern. This global response starts to take effect after a few hours of c-raf induction and affects the whole population of cells after 48 hr. SAHF formation has been shown to occur downstream of several different cellular stresses. The extent of the interactome change, the response time, and the variety of triggers leads us to speculate that this nuclear restructuring may be a fundamental hardwired response of the cell. Our data show a strong correlation between OCI change and isochores; isochores may have physical properties beyond simple sequence recognition that allow the genome to rearrange its architecture during a stress response. A relationship between isochore structure and stress response would have important implications for evolution and could go some way to explaining the differences between the integration and fixation rates seen for some repetitive elements (Costantini et al., 2012).

Senescent cells share similarities with terminally differentiated cells, such as those having permanently exited the cell cycle. Furthermore, effectors of senescence like p53 and p16 have been shown to critically regulate self-renewal in adult stem cells (Janzen et al., 2006). However, there have been few studies on the relationship between senescence and differentiation. The emerging concept of epigenetic rejuvenation aims to reverse the aging process without dedifferentiating target cells (as opposed to iPSC reprogramming), thereby avoiding risks associated with pluripotency, such as cancer. Any realization of epigenetic rejuvenation depends on our ability to discriminate differentiation and senescence (Manukyan and Singh, 2012). ESCs and senescent cells show globally inverted domains of local compaction whereas somatic cells show an intermediate state between ESCs and senescence. Our observations suggest that the remodelling of the higher-order structure we describe for senescence is a continuation of ESC to somatic differentiation. Based on this preliminary observation, reversing these architectural changes may reboot the nuclear architecture in an undifferentiated ESC-like state, contrary to the concept of rejuvenation. However, a deeper understanding of distinct architectural features, distinguishing early differentiation and senescence, could provide a rationale for rejuvenation approaches.

EXPERIMENTAL PROCEDURES

Hi-C

Hi-C was performed essentially as described in Lieberman-Aiden et al. (2009), with some modifications described in the Supplemental Experimental Procedures.

FISH

FISH labeling was performed as described in Bolland et al. (2013). FISH probes were ordered prelabeled from empire genomics. A list of clones can be found in the Supplemental Experimental Procedures. FISH data were analyzed from confocal sections using Volocity software. Top and bottom focal planes were discarded, and the analysis was restricted to central focal planes showing a clear FISH signal. Volocity software was used for automated object detection and distance measurements.

Cell Culture

WI-38hTERT/GFP-RAF1-ER was a generous gift from Carl Mann. Cells were cultured with 10% fetal bovine serum under 5% O2 and handled as described in Jeanblanc et al. (2012). Senescent cells were harvested 48 hr after induction with 4-OH tamoxifen.

Cell Proliferation Assays

BrdU (anti-BrdU; PharMingen) and DAPI staining were carried out as described in Narita et al. (2003).

Computational Data Analysis

Please refer to the Supplemental Experimental Procedures for details about the data analysis.

ACCESSION NUMBERS

The ENA accession number for the sequencing data reported in this paper is PRJEB8073.

SUPPLEMENTAL INFORMATION

Supplemental Information includes Supplemental Experimental Procedures and five figures and can be found with this article online at http://dx.doi.org/10.1016/j.celrep.2014.12.055.

AUTHOR CONTRIBUTIONS

T.C., P.F., and W.R. designed the study. T.C., S.S., M.F.-M., J.-Y.T., and K.K. performed experiments. T.C., P.A.E., S.W.W., and S.A. analyzed the data. T.C., P.A.E., and W.R. wrote the manuscript.

ACKNOWLEDGMENTS

This study was funded by the following grants: BBSRC (BB/K010867/1); BBSRC (BBS/E/B/000C0404); and Wellcome Trust (095645/Z/11/Z). Thanks to Roland Schwarz for useful discussion and to Jesus Gil, Oliver Bischof, David Bazett-Jones, David Gilbert, and Ben Pope for critical reading of the manuscript. Figure icons in the graphical abstract were created by OCHA, Freepik under Creative Commons license 3.0. W.R. is a consultant to Cambridge Epigenetix Ltd.

Received: July 31, 2014
Revised: November 13, 2014
Accepted: December 22, 2014
Published: January 29, 2015

REFERENCES

Acosta, J.C., O'Loghlen, A., Banito, A., Guijarro, M.V., Augert, A., Raguz, S., Fumagalli, M., Da Costa, M., Brown, C., Popov, N., et al. (2008). Chemokine signaling via the CXCR2 receptor reinforces senescence. Cell *133*, 1006–1018.

Acosta, J.C., Banito, A., Wuestefeld, T., Georgilis, A., Janich, P., Morton, J.P., Athineos, D., Kang, T.-W., Lasitschka, F., Andrulis, M., et al. (2013). A complex secretory program orchestrated by the inflammasome controls paracrine senescence. Nat. Cell Biol. *15*, 978–990.

Baker, D.J., Wijshake, T., Tchkonia, T., LeBrasseur, N.K., Childs, B.G., van de Sluis, B., Kirkland, J.L., and van Deursen, J.M. (2011). Clearance of p16Ink4a-positive senescent cells delays ageing-associated disorders. Nature *479*, 232–236.

Banito, A., Rashid, S.T., Acosta, J.C., Li, S., Pereira, C.F., Geti, I., Pinho, S., Silva, J.C., Azuara, V., Walsh, M., et al. (2009). Senescence impairs successful reprogramming to pluripotent stem cells. Genes Dev. *23*, 2134–2139.

Bartkova, J., Rezaei, N., Liontos, M., Karakaidos, P., Kletsas, D., Issaeva, N., Vassiliou, L.-V.F., Kolettas, E., Niforou, K., Zoumpourlis, V.C., et al. (2006). Oncogene-induced senescence is part of the tumorigenesis barrier imposed by DNA damage checkpoints. Nature *444*, 633–637.

Benson, E.K., Lee, S.W., and Aaronson, S.A. (2010). Role of progerin-induced telomere dysfunction in HGPS premature cellular senescence. J. Cell Sci. *123*, 2605–2612.

Bernardi, G. (2012). The genome: an isochore ensemble and its evolution. Ann. N Y Acad. Sci. *1267*, 31–34.

Bolland, D.J., King, M.R., Reik, W., Corcoran, A.E., and Krueger, C. (2013). Robust 3D DNA FISH using directly labeled probes. J. Vis. Exp, e50587.

Braig, M., Lee, S., Loddenkemper, C., Rudolph, C., Peters, A.H.F.M., Schlegelberger, B., Stein, H., Dörken, B., Jenuwein, T., and Schmitt, C.A. (2005). Oncogene-induced senescence as an initial barrier in lymphoma development. Nature *436*, 660–665.

Cao, K., Blair, C.D., Faddah, D.A., Kieckhaefer, J.E., Olive, M., Erdos, M.R., Nabel, E.G., and Collins, F.S. (2011). Progerin and telomere dysfunction collaborate to trigger cellular senescence in normal human fibroblasts. J. Clin. Invest. *121*, 2833–2844.

Chan, H.M., Narita, M., Lowe, S.W., and Livingston, D.M. (2005). The p400 E1A-associated protein is a novel component of the p53 —> p21 senescence pathway. Genes Dev. *19*, 196–201.

Chandra, T., and Narita, M. (2013). High-order chromatin structure and the epigenome in SAHFs. Nucleus *4*, 23–28.

Chandra, T., Kirschner, K., Thuret, J.-Y., Pope, B.D., Ryba, T., Newman, S., Ahmed, K., Samarajiwa, S.A., Salama, R., Carroll, T., et al. (2012). Independence of repressive histone marks and chromatin compaction during senescent heterochromatic layer formation. Mol. Cell *47*, 203–214.

Chicas, A., Wang, X., Zhang, C., McCurrach, M., Zhao, Z., Mert, O., Dickins, R.A., Narita, M., Zhang, M., and Lowe, S.W. (2010). Dissecting the unique role of the retinoblastoma tumor suppressor during cellular senescence. Cancer Cell *17*, 376–387.

Coppé, J.-P., Patil, C.K., Rodier, F., Sun, Y., Muñoz, D.P., Goldstein, J., Nelson, P.S., Desprez, P.-Y., and Campisi, J. (2008). Senescence-associated secretory phenotypes reveal cell-nonautonomous functions of oncogenic RAS and the p53 tumor suppressor. PLoS Biol. *6*, 2853–2868.

Costantini, M., Clay, O., Auletta, F., and Bernardi, G. (2006). An isochore map of human chromosomes. Genome Res. *16*, 536–541.

Costantini, M., Auletta, F., and Bernardi, G. (2012). The distributions of "new" and "old" Alu sequences in the human genome: the solution of a "mystery". Mol. Biol. Evol. *29*, 421–427.

Cruickshanks, H.A., McBryan, T., Nelson, D.M., Vanderkraats, N.D., Shah, P.P., van Tuyn, J., Singh Rai, T., Brock, C., Donahue, G., Dunican, D.S., et al. (2013). Senescent cells harbour features of the cancer epigenome. Nat. Cell Biol. *15*, 1495–1506.

De Cecco, M., Criscione, S.W., Peckham, E.J., Hillenmeyer, S., Hamm, E.A., Manivannan, J., Peterson, A.L., Kreiling, J.A., Neretti, N., and Sedivy, J.M. (2013). Genomes of replicatively senescent cells undergo global epigenetic changes leading to gene silencing and activation of transposable elements. Aging Cell *12*, 247–256.

Di Leonardo, A., Linke, S.P., Clarkin, K., and Wahl, G.M. (1994). DNA damage triggers a prolonged p53-dependent G1 arrest and long-term induction of Cip1 in normal human fibroblasts. Genes Dev. *8*, 2540–2551.

Di Micco, R., Fumagalli, M., Cicalese, A., Piccinin, S., Gasparini, P., Luise, C., Schurra, C., Garre', M., Nuciforo, P.G., Bensimon, A., et al. (2006). Oncogene-induced senescence is a DNA damage response triggered by DNA hyper-replication. Nature *444*, 638–642.

Di Micco, R., Sulli, G., Dobreva, M., Liontos, M., Botrugno, O.A., Gargiulo, G., dal Zuffo, R., Matti, V., d'Ario, G., Montani, E., et al. (2011). Interplay between oncogene-induced DNA damage response and heterochromatin in senescence and cancer. Nat. Cell Biol. *13*, 292–302.

Dixon, J.R., Selvaraj, S., Yue, F., Kim, A., Li, Y., Shen, Y., Hu, M., Liu, J.S., and Ren, B. (2012). Topological domains in mammalian genomes identified by analysis of chromatin interactions. Nature *485*, 376–380.

Eriksson, M., Brown, W.T., Gordon, L.B., Glynn, M.W., Singer, J., Scott, L., Erdos, M.R., Robbins, C.M., Moses, T.Y., Berglund, P., et al. (2003). Recurrent de novo point mutations in lamin A cause Hutchinson-Gilford progeria syndrome. Nature *423*, 293–298.

Funayama, R., Saito, M., Tanobe, H., and Ishikawa, F. (2006). Loss of linker histone H1 in cellular senescence. J. Cell Biol. *175*, 869–880.

Haugstetter, A.M., Loddenkemper, C., Lenze, D., Gröne, J., Standfuss, C., Petersen, I., Dörken, B., and Schmitt, C.A. (2010). Cellular senescence predicts treatment outcome in metastasised colorectal cancer. Br. J. Cancer *103*, 505–509.

Hayflick, L., and Moorhead, P.S. (1961). The serial cultivation of human diploid cell strains. Exp. Cell Res. *25*, 585–621.

Heinz, S., Benner, C., Spann, N., Bertolino, E., Lin, Y.C., Laslo, P., Cheng, J.X., Murre, C., Singh, H., and Glass, C.K. (2010). Simple combinations of lineage-determining transcription factors prime cis-regulatory elements required for macrophage and B cell identities. Mol. Cell *38*, 576–589.

Hiratani, I., Leskovar, A., and Gilbert, D.M. (2004). Differentiation-induced replication-timing changes are restricted to AT-rich/long interspersed nuclear element (LINE)-rich isochores. Proc. Natl. Acad. Sci. USA *101*, 16861–16866.

Hirosue, A., Ishihara, K., Tokunaga, K., Watanabe, T., Saitoh, N., Nakamoto, M., Chandra, T., Narita, M., Shinohara, M., and Nakao, M. (2012). Quantitative assessment of higher-order chromatin structure of the INK4/ARF locus in human senescent cells. Aging Cell *11*, 553–556.

Janzen, V., Forkert, R., Fleming, H.E., Saito, Y., Waring, M.T., Dombkowski, D.M., Cheng, T., DePinho, R.A., Sharpless, N.E., and Scadden, D.T. (2006). Stem-cell ageing modified by the cyclin-dependent kinase inhibitor p16INK4a. Nature *443*, 421–426.

Jeanblanc, M., Ragu, S., Gey, C., Contrepois, K., Courbeyrette, R., Thuret, J.-Y., and Mann, C. (2012). Parallel pathways in RAF-induced senescence and conditions for its reversion. Oncogene *31*, 3072–3085.

Kalhor, R., Tjong, H., Jayathilaka, N., Alber, F., and Chen, L. (2012). Genome architectures revealed by tethered chromosome conformation capture and population-based modeling. Nat. Biotechnol. *30*, 90–98.

Kuilman, T., Michaloglou, C., Vredeveld, L.C.W., Douma, S., van Doorn, R., Desmet, C.J., Aarden, L.A., Mooi, W.J., and Peeper, D.S. (2008). Oncogene-induced senescence relayed by an interleukin-dependent inflammatory network. Cell *133*, 1019–1031.

Li, H., Collado, M., Villasante, A., Strati, K., Ortega, S., Cañamero, M., Blasco, M.A., and Serrano, M. (2009). The Ink4/Arf locus is a barrier for iPS cell reprogramming. Nature *460*, 1136–1139.

Lieberman-Aiden, E., van Berkum, N.L., Williams, L., Imakaev, M., Ragoczy, T., Telling, A., Amit, I., Lajoie, B.R., Sabo, P.J., Dorschner, M.O., et al. (2009). Comprehensive mapping of long-range interactions reveals folding principles of the human genome. Science *326*, 289–293.

Lin, A.W., Barradas, M., Stone, J.C., van Aelst, L., Serrano, M., and Lowe, S.W. (1998). Premature senescence involving p53 and p16 is activated in response to constitutive MEK/MAPK mitogenic signaling. Genes Dev. *12*, 3008–3019.

López-Otín, C., Blasco, M.A., Partridge, L., Serrano, M., and Kroemer, G. (2013). The hallmarks of aging. Cell *153*, 1194–1217.

Macaya, G., Thiery, J.P., and Bernardi, G. (1976). An approach to the organization of eukaryotic genomes at a macromolecular level. J. Mol. Biol. *108*, 237–254.

Manukyan, M., and Singh, P.B. (2012). Epigenetic rejuvenation. Genes Cells *17*, 337–343.

McCord, R.P., Nazario-Toole, A., Zhang, H., Chines, P.S., Zhan, Y., Erdos, M.R., Collins, F.S., Dekker, J., and Cao, K. (2013). Correlated alterations in genome organization, histone methylation, and DNA-lamin A/C interactions in Hutchinson-Gilford progeria syndrome. Genome Res. *23*, 260–269.

Michaloglou, C., Vredeveld, L.C.W., Soengas, M.S., Denoyelle, C., Kuilman, T., van der Horst, C.M.A.M., Majoor, D.M., Shay, J.W., Mooi, W.J., and Peeper, D.S. (2005). BRAFE600-associated senescence-like cell cycle arrest of human naevi. Nature *436*, 720–724.

Muñoz-Espín, D., Cañamero, M., Maraver, A., Gómez-López, G., Contreras, J., Murillo-Cuesta, S., Rodríguez-Baeza, A., Varela-Nieto, I., Ruberte, J., Collado, M., and Serrano, M. (2013). Programmed cell senescence during mammalian embryonic development. Cell *155*, 1104–1118.

Narita, M., Nũnez, S., Heard, E., Narita, M., Lin, A.W., Hearn, S.A., Spector, D.L., Hannon, G.J., and Lowe, S.W. (2003). Rb-mediated heterochromatin formation and silencing of E2F target genes during cellular senescence. Cell *113*, 703–716.

Narita, M., Narita, M., Krizhanovsky, V., Nuñez, S., Chicas, A., Hearn, S.A., Myers, M.P., and Lowe, S.W. (2006). A novel role for high-mobility group a proteins in cellular senescence and heterochromatin formation. Cell *126*, 503–514.

Nora, E.P., Lajoie, B.R., Schulz, E.G., Giorgetti, L., Okamoto, I., Servant, N., Piolot, T., van Berkum, N.L., Meisig, J., Sedat, J., et al. (2012). Spatial partitioning of the regulatory landscape of the X-inactivation centre. Nature *485*, 381–385.

Peric-Hupkes, D., Meuleman, W., Pagie, L., Bruggeman, S.W.M., Solovei, I., Brugman, W., Gräf, S., Flicek, P., Kerkhoven, R.M., van Lohuizen, M., et al. (2010). Molecular maps of the reorganization of genome-nuclear lamina interactions during differentiation. Mol. Cell *38*, 603–613.

Rando, T.A., and Chang, H.Y. (2012). Aging, rejuvenation, and epigenetic reprogramming: resetting the aging clock. Cell *148*, 46–57.

Reimann, M., Lee, S., Loddenkemper, C., Dörr, J.R., Tabor, V., Aichele, P., Stein, H., Dörken, B., Jenuwein, T., and Schmitt, C.A. (2010). Tumor stroma-derived TGF-beta limits myc-driven lymphomagenesis via Suv39h1-dependent senescence. Cancer Cell *17*, 262–272.

Sadaie, M., Salama, R., Carroll, T., Tomimatsu, K., Chandra, T., Young, A.R.J., Narita, M., Pérez-Mancera, P.A., Bennett, D.C., Chong, H., et al. (2013). Redistribution of the Lamin B1 genomic binding profile affects rearrangement of heterochromatic domains and SAHF formation during senescence. Genes Dev. *27*, 1800–1808.

Scaffidi, P., and Misteli, T. (2006). Lamin A-dependent nuclear defects in human aging. Science *312*, 1059–1063.

Serrano, M., Lin, A.W., McCurrach, M.E., Beach, D., and Lowe, S.W. (1997). Oncogenic ras provokes premature cell senescence associated with accumulation of p53 and p16INK4a. Cell *88*, 593–602.

Shah, P.P., Donahue, G., Otte, G.L., Capell, B.C., Nelson, D.M., Cao, K., Aggarwala, V., Cruickshanks, H.A., Rai, T.S., McBryan, T., et al. (2013). Lamin B1 depletion in senescent cells triggers large-scale changes in gene expression and the chromatin landscape. Genes Dev. *27*, 1787–1799.

Shimi, T., Butin-Israeli, V., Adam, S.A., Hamanaka, R.B., Goldman, A.E., Lucas, C.A., Shumaker, D.K., Kosak, S.T., Chandel, N.S., and Goldman, R.D. (2011). The role of nuclear lamin B1 in cell proliferation and senescence. Genes Dev. *25*, 2579–2593.

Shumaker, D.K., Dechat, T., Kohlmaier, A., Adam, S.A., Bozovsky, M.R., Erdos, M.R., Eriksson, M., Goldman, A.E., Khuon, S., Collins, F.S., et al. (2006). Mutant nuclear lamin A leads to progressive alterations of epigenetic control in premature aging. Proc. Natl. Acad. Sci. USA *103*, 8703–8708.

Sofueva, S., Yaffe, E., Chan, W.-C., Georgopoulou, D., Vietri Rudan, M., Mira-Bontenbal, H., Pollard, S.M., Schroth, G.P., Tanay, A., and Hadjur, S. (2013). Cohesin-mediated interactions organize chromosomal domain architecture. EMBO J. *32*, 3119–3129.

Storer, M., Mas, A., Robert-Moreno, A., Pecoraro, M., Ortells, M.C., Di Giacomo, V., Yosef, R., Pilpel, N., Krizhanovsky, V., Sharpe, J., and Keyes, W.M. (2013). Senescence is a developmental mechanism that contributes to embryonic growth and patterning. Cell *155*, 1119–1130.

Swanson, E.C., Manning, B., Zhang, H., and Lawrence, J.B. (2013). Higher-order unfolding of satellite heterochromatin is a consistent and early event in cell senescence. J. Cell Biol. *203*, 929–942.

Thiery, J.P., Macaya, G., and Bernardi, G. (1976). An analysis of eukaryotic genomes by density gradient centrifugation. J. Mol. Biol. *108*, 219–235.

Tu, Z., Zhuang, X., Yao, Y.-G., and Zhang, R. (2013). BRG1 is required for formation of senescence-associated heterochromatin foci induced by oncogenic RAS or BRCA1 loss. Mol. Cell. Biol. *33*, 1819–1829.

Xue, W., Zender, L., Miething, C., Dickins, R.A., Hernando, E., Krizhanovsky, V., Cordon-Cardo, C., and Lowe, S.W. (2007). Senescence and tumour clearance is triggered by p53 restoration in murine liver carcinomas. Nature *445*, 656–660.

Ye, X., Zerlanko, B., Kennedy, A., Banumathy, G., Zhang, R., and Adams, P.D. (2007a). Downregulation of Wnt signaling is a trigger for formation of facultative heterochromatin and onset of cell senescence in primary human cells. Mol. Cell *27*, 183–196.

Ye, X., Zerlanko, B., Zhang, R., Somaiah, N., Lipinski, M., Salomoni, P., and Adams, P.D. (2007b). Definition of pRB- and p53-dependent and -independent steps in HIRA/ASF1a-mediated formation of senescence-associated heterochromatin foci. Mol. Cell. Biol. *27*, 2452–2465.

Young, A.R.J., Narita, M., Ferreira, M., Kirschner, K., Sadaie, M., Darot, J.F.J., Tavaré, S., Arakawa, S., Shimizu, S., Watt, F.M., and Narita, M. (2009). Autophagy mediates the mitotic senescence transition. Genes Dev. *23*, 798–803.

Zhang, R., Poustovoitov, M.V., Ye, X., Santos, H.A., Chen, W., Daganzo, S.M., Erzberger, J.P., Serebriiskii, I.G., Canutescu, A.A., Dunbrack, R.L., et al. (2005). Formation of MacroH2A-containing senescence-associated heterochromatin foci and senescence driven by ASF1a and HIRA. Dev. Cell *8*, 19–30.

Cell Reports

Supplemental Information

Global Reorganization of the Nuclear Landscape in Senescent Cells

Tamir Chandra, Philip Andrew Ewels, Stefan Schoenfelder, Mayra Furlan-Magaril, Steven William Wingett, Kristina Kirschner, Jean-Yves Thuret, Simon Andrews, Peter Fraser, and Wolf Reik

Extended experimental procedures

Hi-C
Cells were fixed in 2% formaldehyde for 10 minutes. After digestion with HindIII (NEB) overnight at 37˚C, restriction sites were filled in with Klenow (NEB) using biotin-14-dATP (Life Technologies). Ligation (4 hours at 16˚C) was followed by reversal of crosslinks (65˚C overnight in the presence of Proteinase K), RNase A treatment, and phenol chloroform extraction of ligation products. The concentration DNA was determined using Quant-iT Pico Green (Life Technologies), and 25 µg of Hi-C library DNA were subjected to removal of biotin from non-ligated fragment ends, followed by sonication (Covaris E220). Sonicated DNA was end-repaired and size selected using AMPure XP beads (Beckman Coulter), before dATP-tailing with Klenow exo- (NEB). Biotin-marked ligation products were isolated using MyOne Streptavidin C1 Dynabeads (Life Technologies), and Tru-seq adapters (Illumina) were ligated onto Hi-C ligation products bound to streptavidin beads. Bead-bound Hi-C DNA was amplified with rounds of 9 PCR amplification. Hi-C libraries were interrogated by paired-end sequencing.

FISH clones:
RP11-937K23 chr18:63788654-64008278
RP11-104N14 chr18:44546277-44745145
RP11-953M14 chr9:24147300-24351523

Chromosomal interaction heatmaps
Heatmaps were generated using HOMER as described in documentation (Heinz et al., 2010). Results were viewed using Java Tree View (Saldanha, 2004).

WashU Interaction Plots
Interaction BED files were generated using WashU_Pipe and viewed on the WashU Epigenome Browser (Zhou et al., 2013). WashU_Pipe is a tool to convert BAM/SAM files generated by HiCUP into interaction BED files in a format that can be interpreted by the WashU Epigenome Browser. The absolute number of interactions between two bins determines the strength of a given interaction. WashU_Pipe can create data subsets so that only interactions between a region of interest and the remainder of the genome are displayed. Interaction counts were binned into 40kb windows and thresholds were set according to overall read counts from the libraries to normalise for sequencing depth.

TAD internal / external interactions
"Read count" and "HiC read count" quantitations were used in SeqMonk (http://www.bioinformatics.babraham.ac.uk/projects/seqmonk/), using the selected TAD as a single probe. HiC read count ignores second read pair count if within the same window, so difference gives TAD-internal interactions. "4C other ends" data import was used to gather all ditag other ends from selected TAD. Read counts were generated for the remaining parts of the chromosome and all trans chromosomes. All values were normalised for total HiC read counts between samples.

Calculation of OCI
OCI values were calculated using SeqMonk. Rolling windows were created across the genome (200kb unless otherwise stated). Raw HiC ditag counts were calculated for each window; those with no counts or extremely high counts were removed. The "cis/trans" quantitation method was then used on samples. The chromosomal median values were subtracted for all windows. The "smoothing subtraction quantitation" using a window size of 20 megabases was used to normalise for biases along the lengths of the chromosomes.
For *cis*-only OCI, *trans* reads were discarded and the "use distal reads as trans" option was used in the "cis/trans" SeqMonk quantitation, taking reads >= 20mb away as distal.

DNase hypersensitivity scatter plot
DNase hypersensitivity data was downloaded from the UCSC web browser for IMR90 cells. This was trimmed and aligned using bowtie. Reads were imported into SeqMonk and counted in the same windows used for OCI calculation. Reads counts were exported before being log transformed in R. Data was thresholded to remove extreme values and used to colour scatter plot points.

%GC Content scatter plot
The %GC content of genomic windows used for OCI scatter plot was calculated using a custom script. Data was thresholded to remove extreme values and used to colour scatter plot points.

Isochores
Isochore co-ordinates were taken from (Costantini et al., 2006). LAD – isochores were used based on their overlaps. If a LAD spans two isochores it is split, with the exception of Fig S2 where LADs were classified into isochores based on the greatest overlap.

TAD cross-boundary ratios
Counts of ditags within TADs and spanning TAD boundaries were calculated using SeqMonk with the "read count" and "HiC read count" method described above. TADs were assigned to isochores and plotted in R.

Identification of Opening and Closing TADs
Variation of the insulation of all TADs was calculated by assuming the true mean to be 0 (no difference between growing and senescence). Mean and difference between insulation scores were calculated for each point. Data was ordered by means and the standard deviation was calculated for each point using the 50 points ahead and behind it. Assuming the mean to be 0 and the data to have a normal distribution, p values were calculated using the pnorm function.

Opening TADs were called by selecting only points with $p < 0.05$. As this cutoff did not select any closing TADs, a second cutoff ($p < 0.25$) was used and filtered for only closing TADs for comparison.

Feature enrichment
Expected overlap per bp was calculated for each feature type (total length of feature / genome length). Observed scores were calculated for the overlap of each feature (overlap between region of interest and feature / total length of region of interest). Enrichment scores were calculated with $\log_2$ of (observed / expected).

Change in OCI
Change in OCI was calculated by subtracting senescence OCI scores from growing for each genomic window.

Change in interaction strengths
Interaction counts were calculated using the "4C other ends" feature in SeqMonk . Ditag ends were taken from all regions marked as a feature of interest. Other ends were ignored if falling within the same feature bin as their partner or if their partner was within 2 megabases. Overlap of resulting other ends was calculated with simple read count quantitation. Values were normalised according to total HiC read counts.

Changes in interaction were calculated by subtracting the growing scores from senescence.

Change in replication timing
Replication timing data was downloaded from http://www.replicationdomain.com/ (ChipID: 369858-3 IMR90 and 508590-2A03 IMR90 Ras). Co-ordinates were converted to hg19 genome build using the niceLiftOver tool (https://github.com/ewels/niceLiftOver). Data was binned into 200kb windows and plotted in R. Original smoothed data (unbinned) was used for the browser shot.

Hierarchical clustering
OCI was calculated using 1 megabase windows due to poor quality of progeria HiC data. Hierarchical clustering was performed using SeqMonk. %GC content of probes was calculated using a custom script and plotted using R.

Conservation of ES OCI-domains
OCI data was discretised in 200kb windows as distal or local according to whether it had a positive or negative score. OCI domains were called using a hidden markov model in R. The percentage of bins that were distal within each domain was called for the discretised ES, growing and senescent data. ES data follows a bimodal distribution of being nearly all either distal or local, as expected.

References

Costantini, M., Clay, O., Auletta, F., and Bernardi, G. (2006). An isochore map of human chromosomes. Genome Res. *16*, 536–541.

Heinz, S., Benner, C., Spann, N., Bertolino, E., Lin, Y.C., Laslo, P., Cheng, J.X., Murre, C., Singh, H., and Glass, C.K. (2010). Simple combinations of lineage-determining transcription factors prime cis-regulatory elements required for macrophage and B cell identities. Mol. Cell *38*, 576–589.

Saldanha, A.J. (2004). Java Treeview--extensible visualization of microarray data. Bioinformatics *20*, 3246–3248.

Zhou, X., Lowdon, R.F., Li, D., Lawson, H.A., Madden, P.A.F., Costello, J.F., and Wang, T. (2013). Exploring long-range genome interactions using the WashU Epigenome Browser. Nat. Methods *10*, 375–376.

Figure S1 (related to Fig 1)

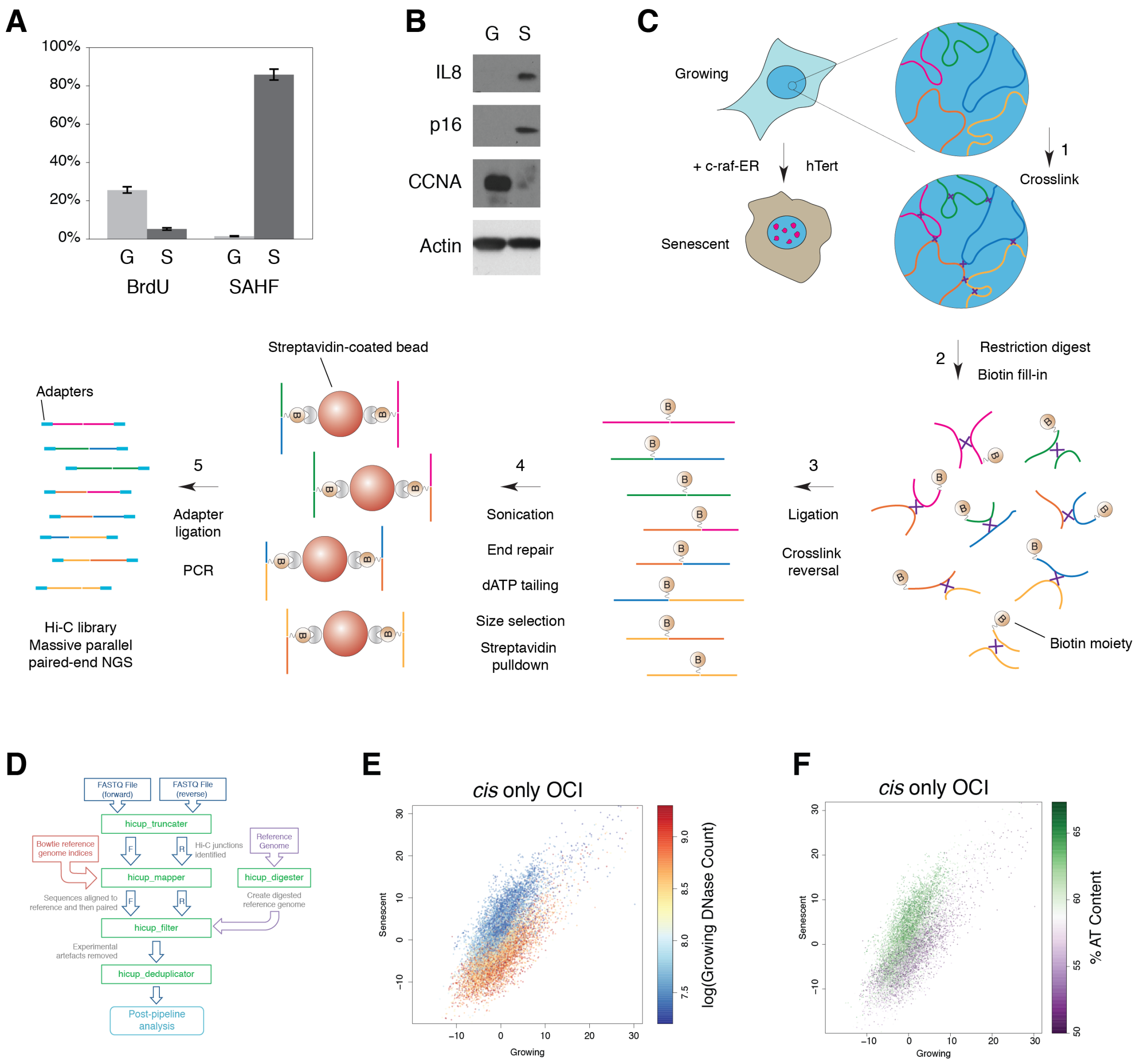

Senescence is accompanied by local and global changes in the interaction pattern of the genome (related to Figure 1).

(A) BrdU and SAHF count for growing (G) and senescent cells (S). (**B**) Western blot of key senescent markers between growing (G) and senescence (S) from whole cell lysate. **(C)** Diagram of Hi-C experimental work-flow. Cells were fixed in formaldehyde to preserve the nuclear architecture. Chromatin was digested with HindIII and restriction sites were filled in using biotinylated dATPs. The following ligation captured spatially proximal ends. Biotin-marked ligation products were enriched, amplified and sequenced. **(D)** Flow diagram showing HiCUP HiC processing data flow. **(E)** Scatter plot for OCI values calculated in cis. DNase accessibility is highlighted according to colour scale. **(F)** Scatter plot for OCI values calculated in cis. AT content is highlighted according to colour scale.

Figure S2 (related to Figs 2 and 3)

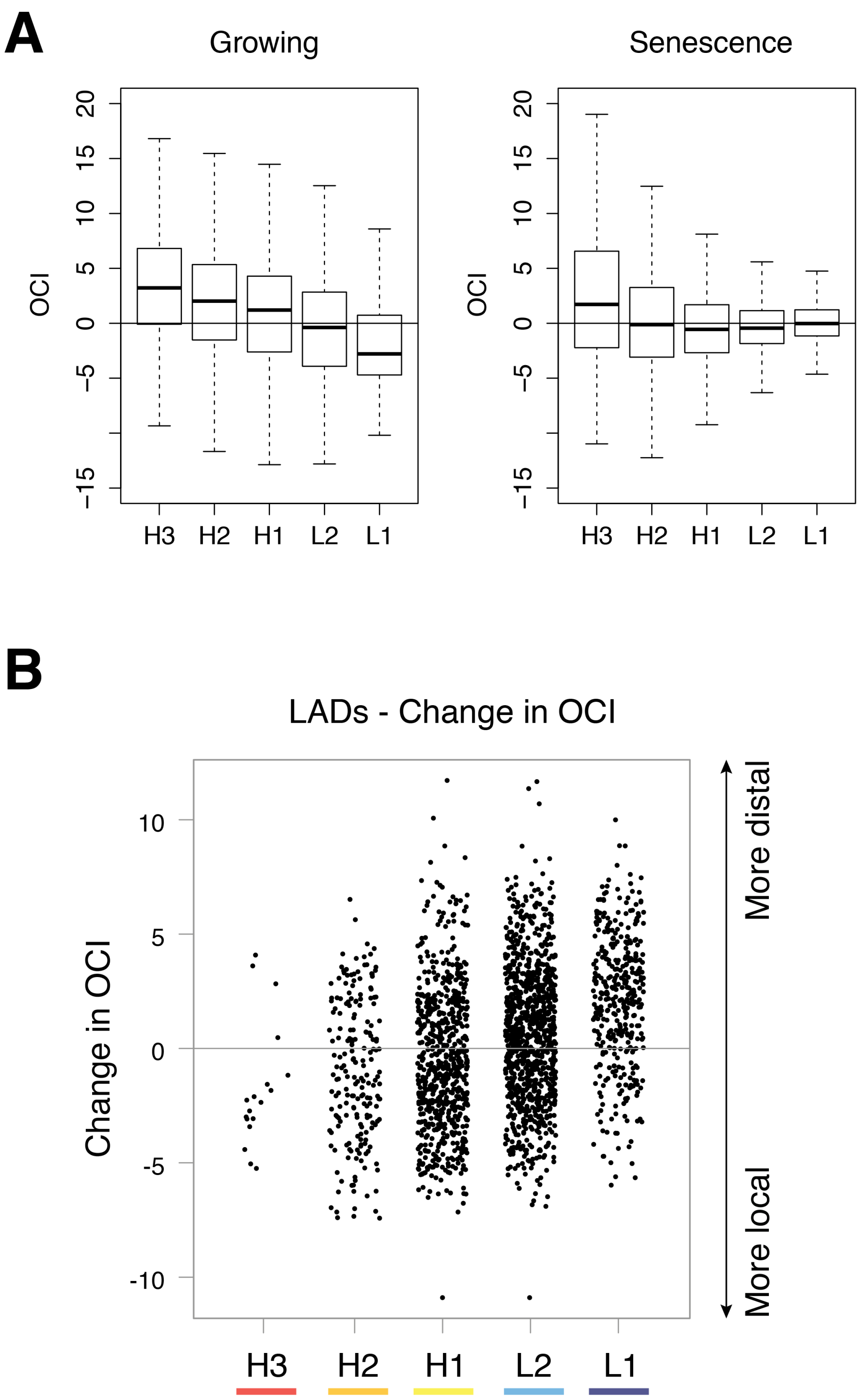

Sequence composition predicts structural chromatin dynamics in senescence (related to Figures 2 and 3)

(A) Boxplots showing distribution of OCI across different isochores in Growing and Senescent cells. **(B)** Strip-chart showing change in OCI in LADs. Each point is the average for an individual LAD.

Figure S3 (related to Fig 4)

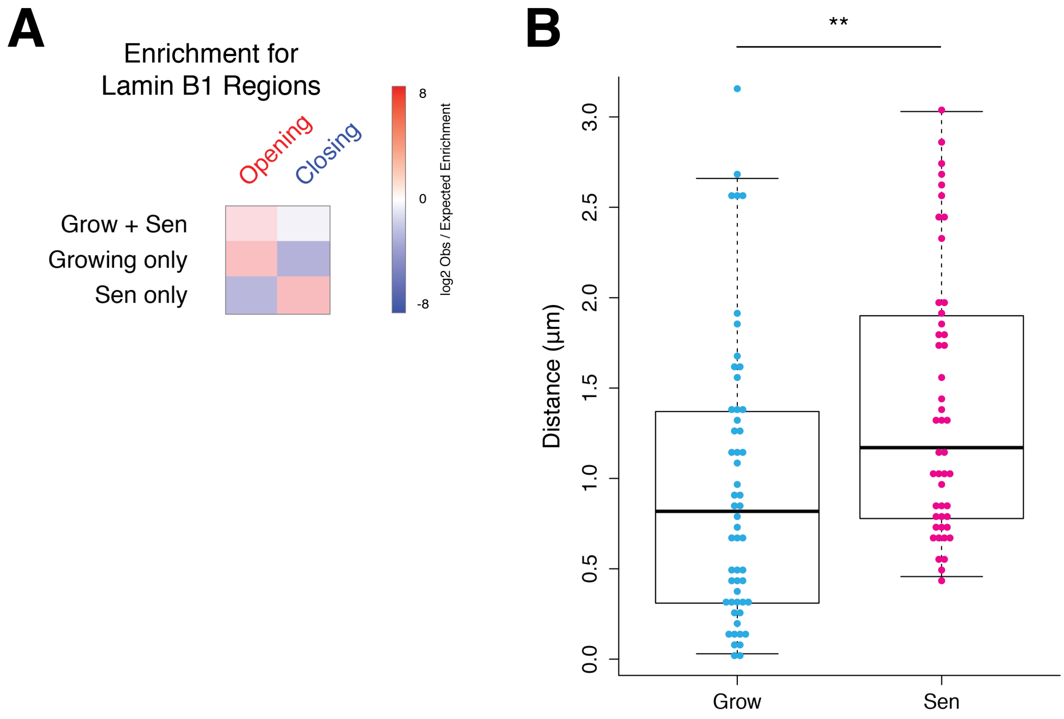

Combined sequence composition and Lamin association predict the strongest OCI gaining regions, which are changing their nuclear positioning (related to Figure 4).

(A) Enrichment of opening and closing TADs for LMNB1 regions found in growing and senescence (Grow + Sen), only in growing (Growing only) or only in senescence (Sen only). **(B)** Distance to periphery for a L1 LAD in Growing and Senescence ($p < 0.002$; Wilcoxon Mann Whitney test). Probe: RP11-953M14, chr9:24147300-24351523

Figure S4 (related to Fig 6)

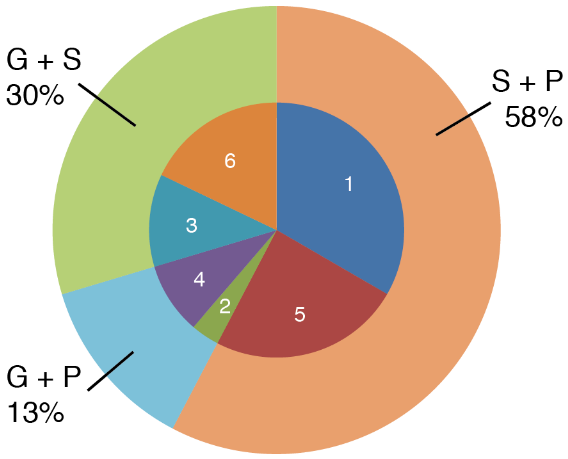

Higher order chromatin dynamics in senescence reflect changes in progeria and may represent the end point of a continuous remodelling process in differentiation (related to Figure 6).

Genome percentages of clusters from hierarchical clustering from Figure 6. The inner circle shows the genome percentage of individual clusters. The outer circle shows where growing and senescence (G+S), senescence and progeria (S+P) and growing and progeria (G+P) show similar trends in OCI.

Figure S5 (related to Fig 6 and Discussion)

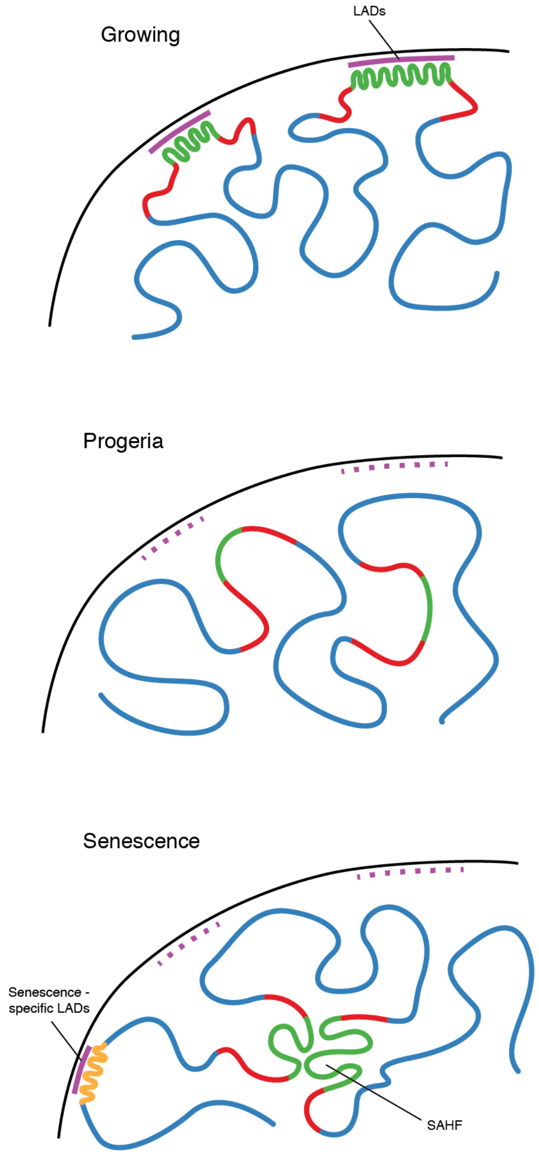

A model reconciling observations from cellular senescence and progeria (related to Figure 6 and Discussion).

Model comparing SAHF formation and HGPS. The destabilisation of LAD heterochromatic areas is common to SAHF formation and HGPS cells. SAHF formation shows a specific clustering of these heterochromatic domains.
